# Improving radiation therapy efficacy considering DNA repair, TP53 mutations, microscopic heterogeneity, and low- and high-dose apoptosis

**DOI:** 10.3389/fonc.2025.1703503

**Published:** 2026-03-02

**Authors:** Anders Brahme

**Affiliations:** Department of Oncology-Pathology, Karolinska Institutet, Stockholm, Sweden

**Keywords:** TP53 damage sensors and modification sites, multiply damaged sites, dual nucleosomal double-strand breaks, low-dose hyper-sensitivity, low-dose apoptosis, light ion radiation therapy, therapy optimization, optimal daily-weekly fractionation

## Abstract

All radiation types produce δ -rays of about a ≈1 keV or less that can impart MGy doses to 10-nm-size volumes of DNA. These events can produce severe dual double-strand breaks (DDSB) at the periphery of nucleosomes in single events particularly in heterochromatic DNA. These DDSBs are the most common multiply damaged sites, and their probabilities generally determine the biological effectiveness and therapeutic responses. The recent understanding that most normal tissues with intact TP53 genes generally are low-dose hypersensitive (LDHS) and low-dose apoptotic (LDA) implies that the well-known universal clinical fractionation window at ≈2 Gy/Fr defines the optimal tolerance level of most organs at risk and not the optimal tumor dose per fraction at least when using intensity-modulated radiation therapy (IMRT). Interestingly, practically all cancer cells are linked to genomic instability in some DNA repair, cell cycle, or growth control genes like TP53 that is affected in more than 50% of all tumors. Unfortunately, this often gives tumor cells a low-dose radiation-resistant (LDRR) phenotype. The fractionation window is due to the low-dose and linear energy transfer (*LET*) initiation of full DNA repair capability after ≈½ Gy or 18 DSB, and we should use this acquired repair advantage in normal tissues to its full extent up to ≈2.3 Gy where the high-dose apoptosis (HDA) starts to set in. Understanding quantum biological cure implies that light ions should truly have the lowest possible *LET* in normal tissues to retain the classical fractionation window but have a high *LET* only in the gross tumor region. Carbon ion therapy substantially benefits from the last ≈10 GyE of the treatment being delivered by low *LET* (electrons or photons) to minimize normal tissue damage, get a steepest possible dose response, and maximize complication-free cure. Interestingly, this also necessitates the use of the lightest ions with a low *LET* in normal tissues, allowing quantum biology-optimized molecular radiation therapy with He-Li-B ions, with minimal adverse therapeutic effect in normal tissues and the highest possible apoptosis, senescence, and cell kill in the tumor!

## 1 Introduction

**Figure 1:**
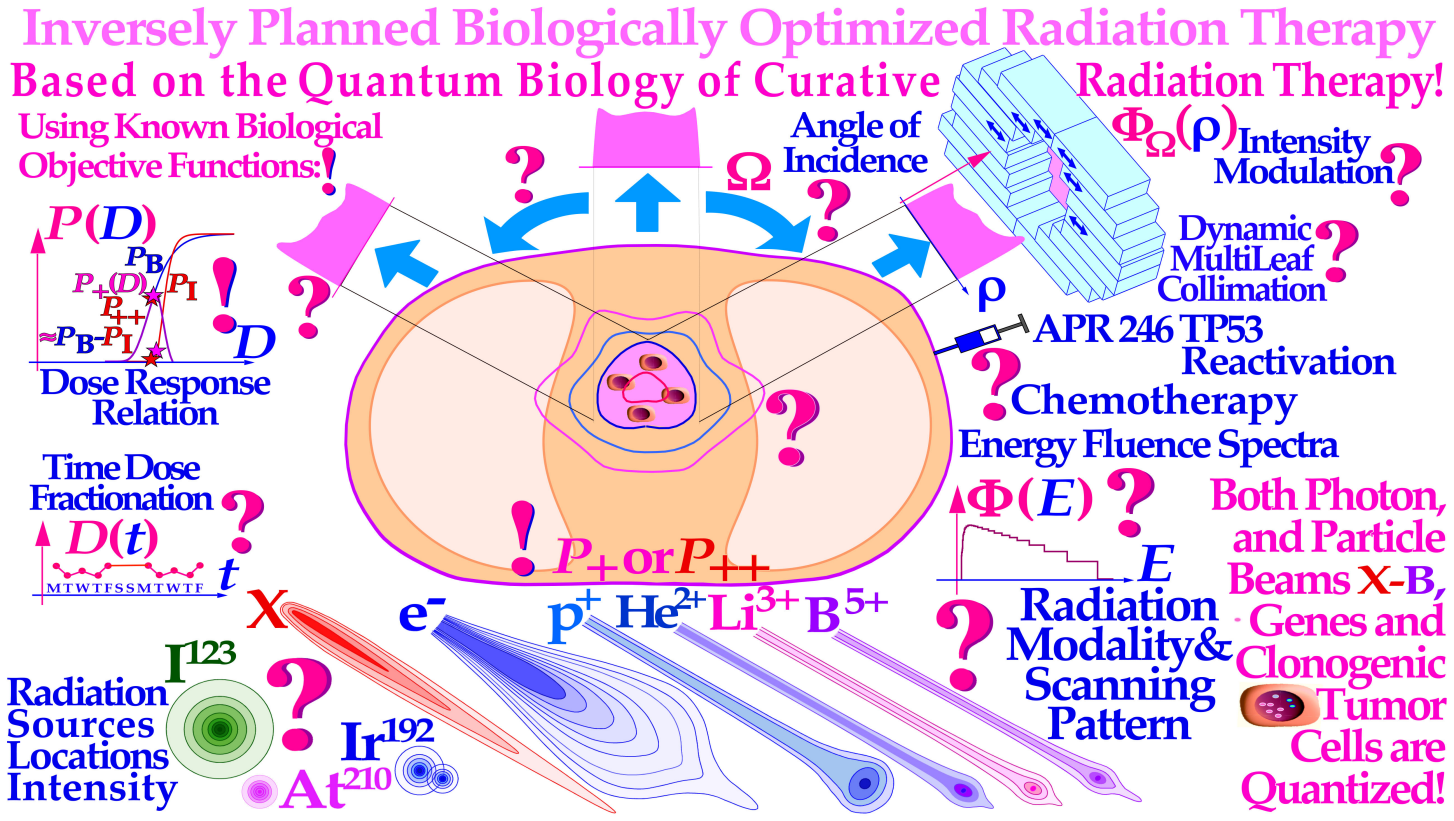
There is a fantastic power of curability available by employing biologically optimized inversely planned radiation therapy, where the intensity of each pencil beam can be directed and modulated to maximize the complication-free cure probability for the patient with minimal risk for side effects in normal tissues (1–4). If the approximate sensitivity of the tumor can be determined from its response, the first week of therapy and the normal tissue responses are generally quite well known from historical data [!; see [2].: Figures 113, 123–128 (2, 5, 6, 17, 19-21, 27)], and it is possible to derive the biologically optimal beam directions and their intensity modulation by dynamic multi leaf collimation or scanning pencil beams (? (3, 4);). It is even possible to find the optimal combination of low and high ionization density radiation (cf. Figures 10–11 [2]: Figures 103–108, 118) and their incident energy spectra as well as the ideal time dose fractionation pattern (as seen in **Figure 12** (2, 7, 8) (9);: Figures 78– 84) using the biologically optimized complication-free cure (P+) or more advanced treatment optimization strategies [P++: P+ followed by concomitant constrained injury minimization (3, 4, 10, 11)]. The figure is modified from (9): **Figure 4** with permission.

Radiation therapy is the most curative treatment method for many cancers, and it works through the genomic instability that makes tumor cells more vulnerable to DNA damage than intact normal tissues, and its therapeutic effect can be readably optimized as indicated in **Figure 1**. Interestingly, there is genomic instability in practically all cancer cells as some DNA repair, cell cycle, and growth control genes are generally mutated, such as TP53, which is affected in more than 50% of all tumors (1, 7–9, 12–14). This generally makes tumor cells more vulnerable to high-dose DNA-damaging agents than intact normal tissues, often due to lack of apoptosis, cell cycle blockade, and lack of high-fidelity DNA repair often via their TP53 mutations. Unfortunately, they are at the same time commonly associated with a low-dose radiation-resistant (LDRR) phenotype (cf. Figures 8 and 9 [1, 7]), requiring higher tumor doses per fraction for cure. Recent developments in nanometer resolution DNA damage imaging have identified the key effector of curative radiation therapy as the dual double-strand breaks (DDSBs) on nucleosomes by low-energy δ-rays in both ion, electron and photon beams (9, 15, 16) and make new ways possible to optimize radiation treatments. Interestingly, this makes the DDSB produced by δ-rays one of our smallest and most effective pills or drugs against genomic instable tumors (9, 17–19).

A key question in this paper is, thus, how to deliver these effective δ-rays safely only to the tumor cells. One answer is very simple: Use advanced molecular radiation biologically optimized inversely planned electron, photon, and light ion radiation therapy! The most effective method will be molecular radiation therapy with the unique lithium ions, as they combine low ionization density with easily repairable damage everywhere except in the tumor where the highest possible apoptosis and senescence is induced in a ≈5-mm-diameter volume around the Bragg peak [cf. lower lane of Figure 8 (1, 5, 7, 8)]. Thus, they are keeping the unique and important daily fractionation window of classical low linear energy transfer (*LET*) electron and photon beam therapy fully open (1, 7, 9, 10).

With the lightest ions above protons, He-B, the clinical border region between the gross tumor and the associated internal target volume due to tumor and tissue motions in relation to surrounding healthy normal tissues can be set as narrow as physically possible as seen in **Figure 1** [cf. also Figure 8 (3, 4, 8)]. In addition, the optimal number of treatment fractions can be substantially reduced, and the initial curative gain factor on massive radiation-resistant hypoxic tumors can often be more than doubled compared to low-ionization-density photons, electrons, and protons not least when high-resolution molecular tumor imaging is available (2, 4). The method in **Figure 1** applies mainly to the most advanced treatment units with scanned electron, photon, or ion beams and/or dynamic multileaf collimation (11, 20–23). However, it will also show for more simple treatment units how the optimal selection of therapeutic beams can be arranged and the intensity and energy modulation be shaped to maximize the complication-free cure probability for the patient with minimal risk for side effects in normal tissues (1, 3, 4, 10). The present paper is kind of an introduction to an open access book on Fundamental Molecular Understanding of Quantum Biology Optimized Curative Radiation Oncology (9) where many of the new ideas presented here are discussed and illustrated in much further detail based on the present key references. During the last week of conventional curative treatment, only a handful of viable tumor clonogens remain in the target volume, as indicated in **Figure 1** [cf. also Figures 10–14] and they need to be treated with the utmost care as most of the initial internal target volume is almost inactivated and the much more microscopically uniform and homogeneous electron or photon beams largely without microscopic cold spots are a necessity for effective cure (cf. Figures 1, 11, and 12 and [7]: Figures 67–71). In particular, ion beams and electron and photon beams and the tumor cells with their individual genes are quantized, and thus, some tumor clonogens may be protected from lethal hits by the inevitable Poissonian cold spots between the ion paths. During the last few and normally most curative therapeutic dose fractions, they are generally lost with Poissonian random ion beams (cf. Figures 10–12 and [7]: section 5)! Electron or photon beams have therefore the steepest possible dose response and simultaneously less normal tissue damage and can thus make a true maximization of the complication-free cure, as clearly demonstrated in the present study [cf. Figures 11–14 and sections 5 and 6 (1, 3, 5, 7, 8, 12)].

## 2 Radiation energy deposition and the biological effectiveness of DDSBs

**Figure 2:**
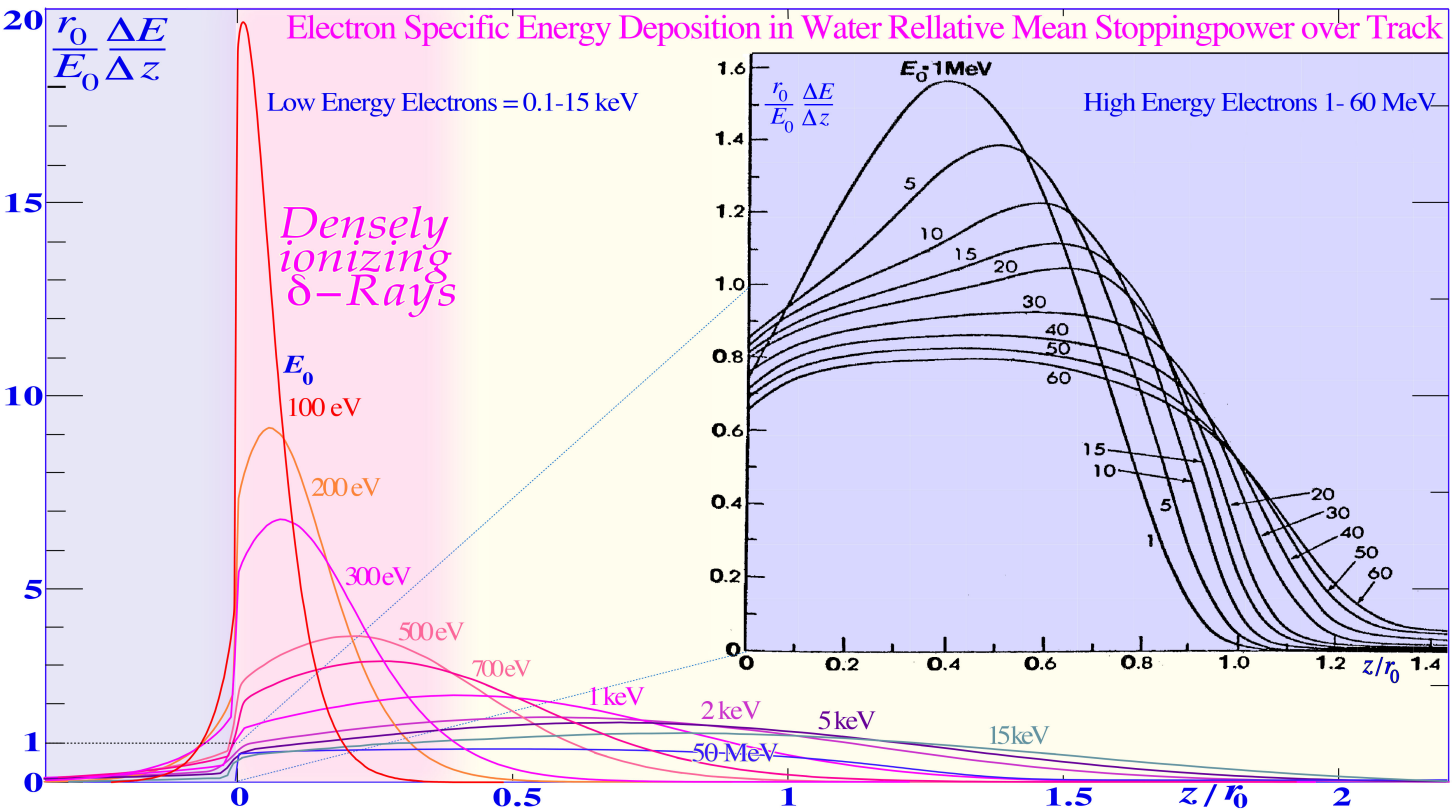
The mean energy deposition in plane parallel electron beams and typical individual electron tracks, scaled by the continuous slowing down range *r*_0_ and the mean stopping power *E*_0_/*r*_0_ over the continuous slowing down range (cf [25].: Figures 2.21–2.22). The vertical dose scale is magnified almost 10 times for the high-energy electrons to the right that have an almost constant effective stopping power of ≈2 MeV/gcm^-2^ ≈ 0.2 eV/nm. Therefore, with minimal damage to DNA, something only their sub-keV secondary track ends can do, as seen to the right in the figure by a ≈20-fold increase in specific energy (pink shading, cf. Figures 3 and 4, modified from [7, 26–28]). The most efficient source of such high “*LET* δ −electrons” are the light ions as seen by their slowing down spectra (cf [7].: Figures 15–19). Modified with permission from [7: Figure 22].

**Figure 3:**
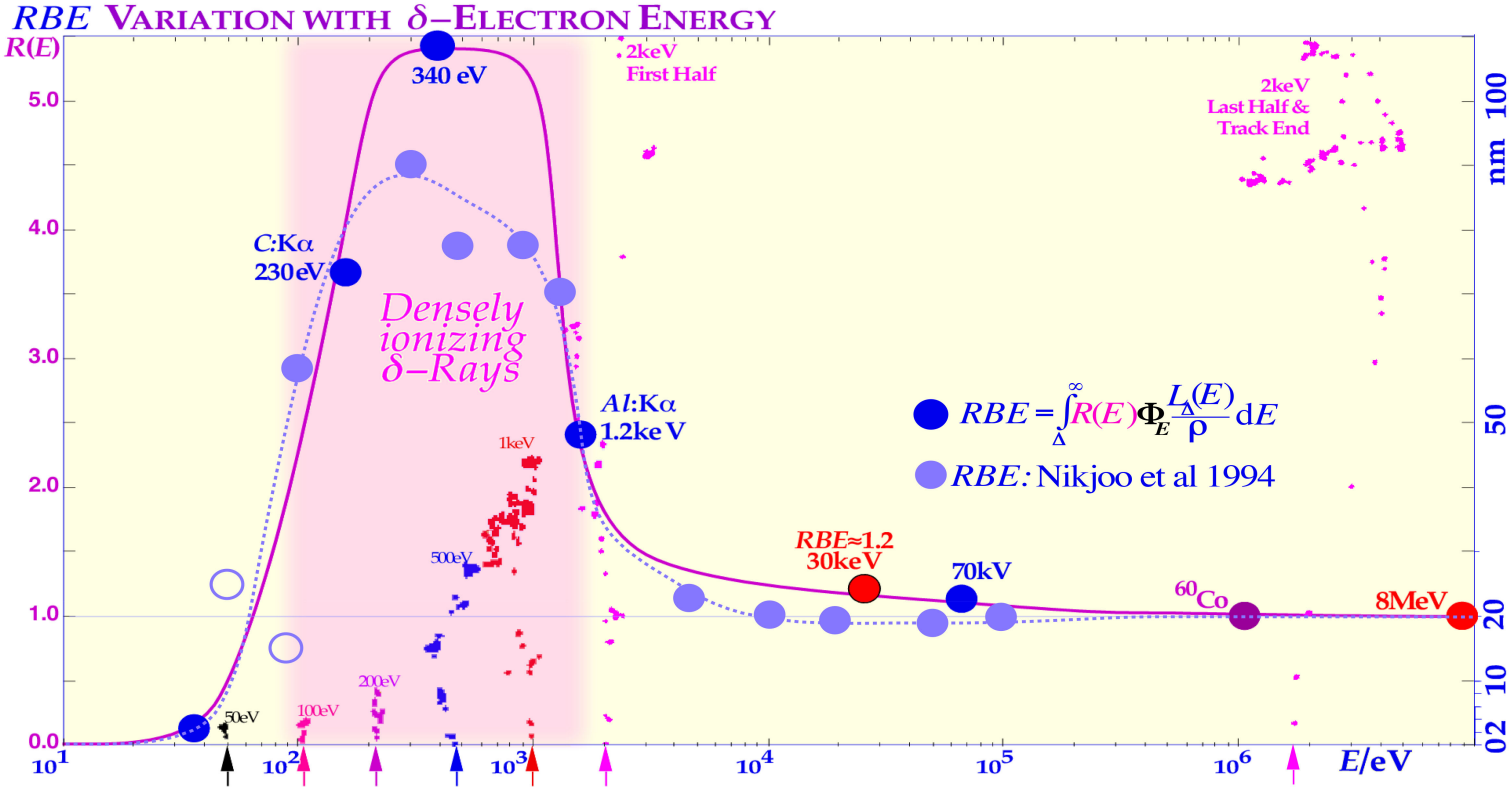
The high relative biological effectiveness (RBE) of secondary electron slowing down spectra of sub-keV and higher-energy electrons and photons in water. The pink regions show where a high fluence of δ-rays are generated, either in low-keV electron or in photon beams (cf [7].: Figures 15–19). The high scattering power, with multiple scatter detours, secondary electron production,and basically medium-high *LET* (25 eV/nm [7]: cf. Figures 15-19), makes the low-energy δ-rays truly high *LET*-like (≈50 eV/nm, cf. Figure 4 upper right corner [28–31]! The figure is modified with permission from [7: **Figure 16**]).

**Figure 4:**
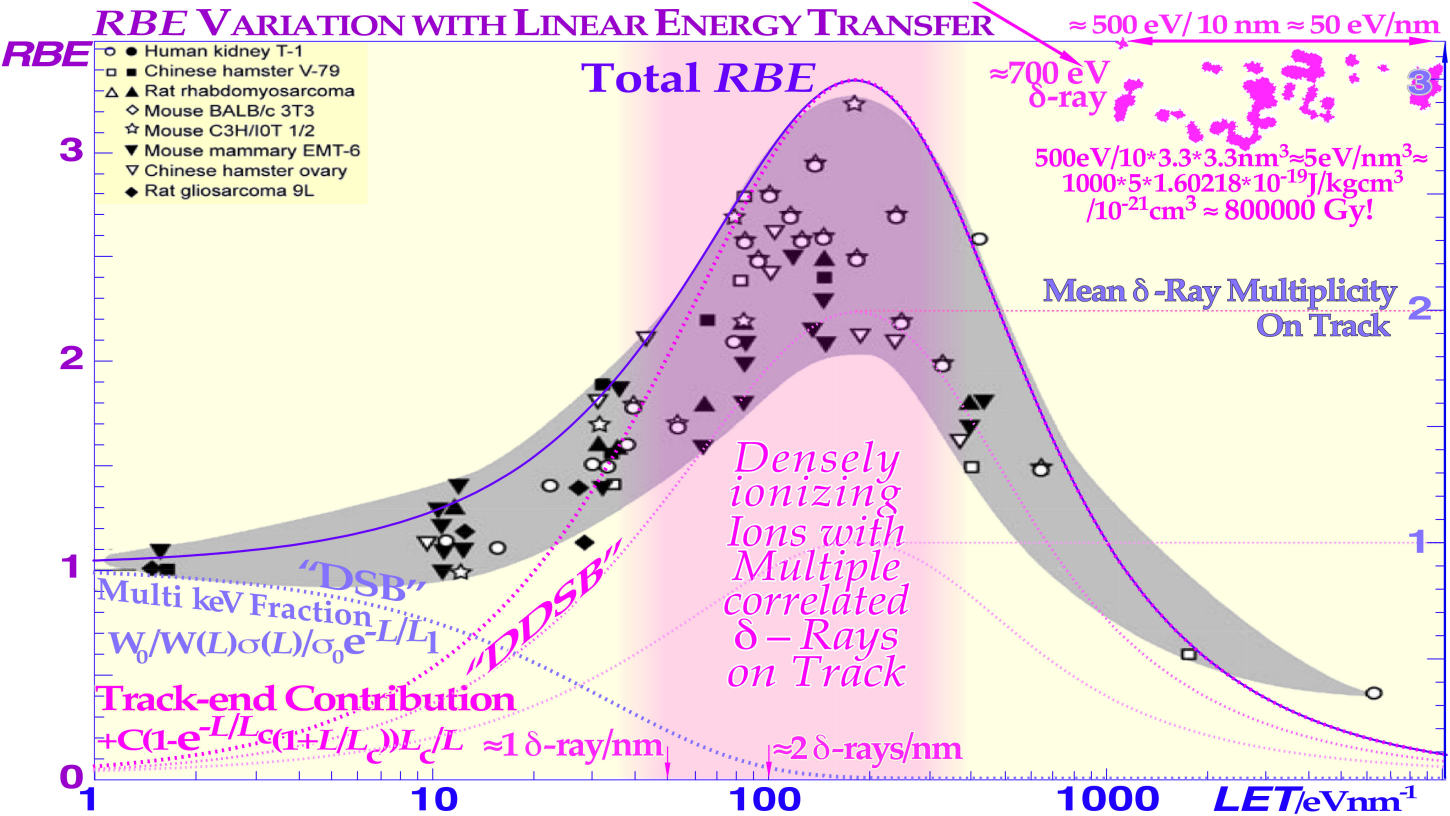
As the inactivation cross-section saturates (cf [7].: Figure 91, 97), the peak biological effectiveness or *RBE* appears since the cross-section cannot increase with the *LET* any more, and at higher *LET*s, the biological effectiveness decreases because of a quasi-constant cross-section and increasing probability of radical–radical recombination as secondary electrons are generated more and more closely together at narrow radii, so “overkill” and recombination effects sets in (cf [7].: Figures 91, 92, and 97). The dashed curves are taken from (24–27) and describe the average response of the multiple experimental data sets very well by a dual Poisson process [a miss and a single DSB hit generally leads to survival, cf [7].: Eq. (5) and Figures 17 and 94, modified from (24–27) with permission].

**Figure 5:**
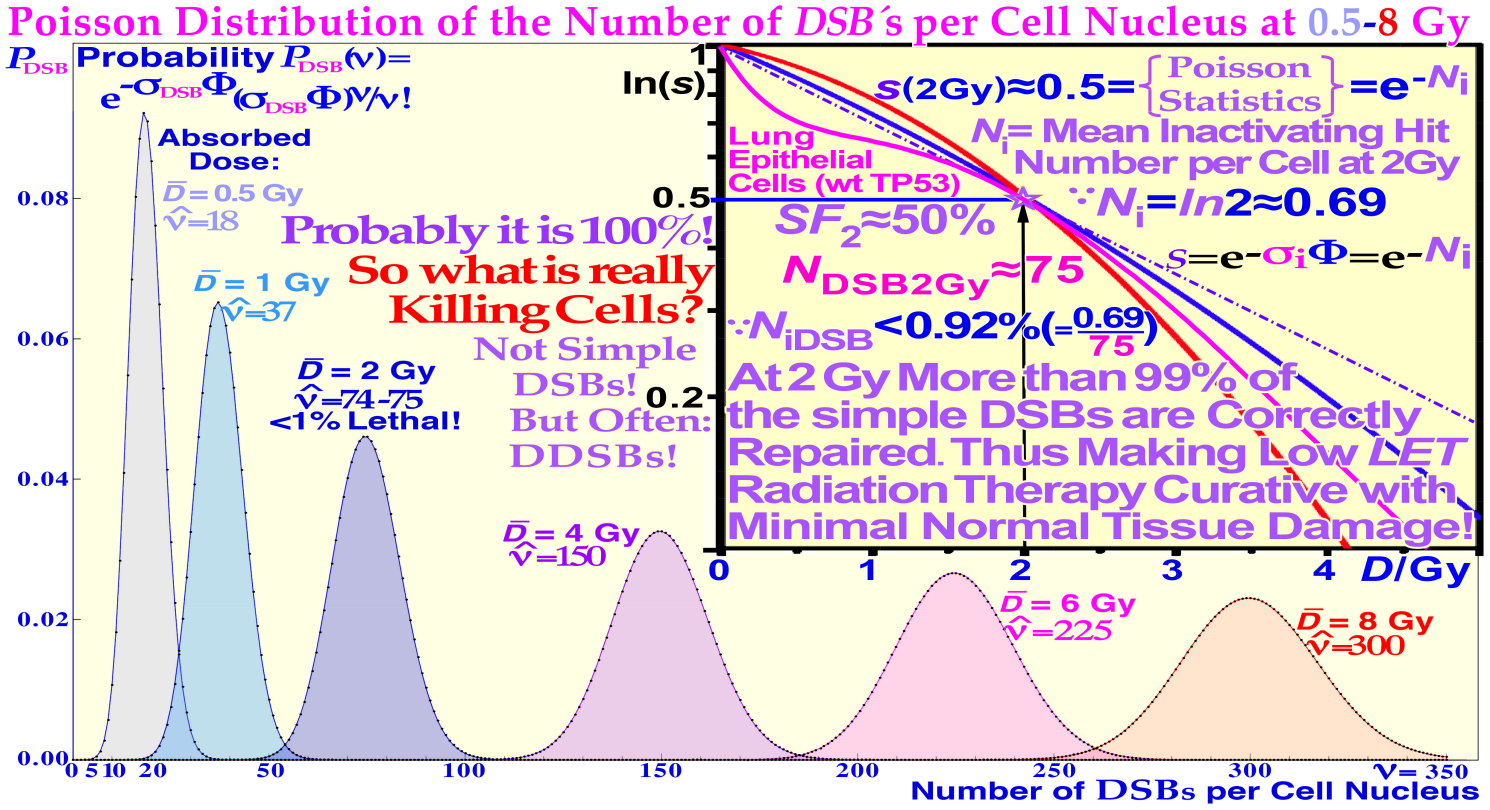
The induction DSBs and repairosomes as a function of dose and *LET* will largely follow Poisson statistics at least up to some 5–10 Gy and 100 eV/nm. At approximately 2 Gy, less than 1% of the DSBs are lethal, as calculated in the middle insert [modified from (9) (10);: **Figures 1** and **2** and Eqs. (9–11)]. The right inserts show the structure of high *LET* ion and photon-induced cellular repairosomes or repair foci at a few Gy, and a cell nuclear width ≈10 μm. Interestingly, 2 Gy low *LET* radiation is almost at the limit of producing a lethal event in normal tissues; thus, a dose at the upper border of the fractionation window of normal tissues and higher should be reserved only for tumor tissue (cf Figures 8 and 18 below) (9): Figures 47–53, 58, and 78. **Figure 6** shows that the 0.34+0.25 ≈ 0.69 potential killing events at 2 Gy are most likely due to the much more severe but fewer δ -electron track end generated DDSBs as discussed in detail in (15) (9);: sections 4.9–4.11.

**Figure 6:**
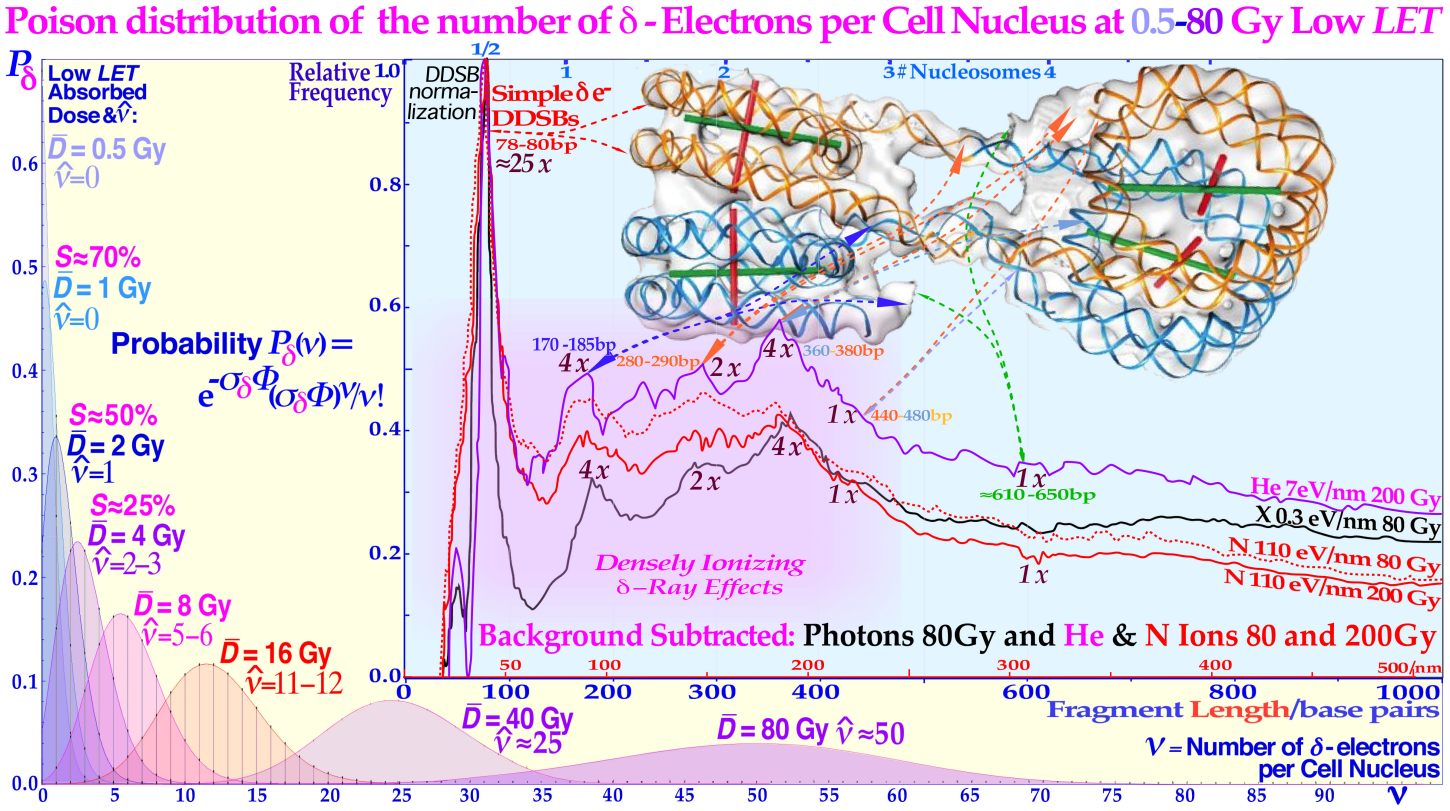
The Poisson distribution of the number of δ -electrons per cell nucleus at doses around 0.5–80 Gy low *LET*. A common DDSB event by a single δ -electron is also indicated, delivering approximately ½–1 MGy in a few hundred nm^3^ local volume at the periphery of a nucleosome (cf. Figures 2–4), and the risk is even higher in the di- or tetra-nucleosomal configuration found in heterochromatin (9, 15, 27, 44–46). The insert gives an overview of the δ-electron production and resultant induced DDSBs (at ≈80 bp or 40 nm) and DNA fragment length distributions generated after irradiation with photons and light ions and shows the great similarity between them here with their spontaneous background distribution subtracted (9, 34). The DNA fragment distribution reminds us of the ladder formation during apoptosis of nucleosomal DNA but is here largely produced by the short reach of the sub-keV δ-electron track ends ≈10–15 nm (cf. Figures 3 and 4, upper right corner). Interestingly, they are sure to be always all alone in the ≈50-nm tetra-nucleosome unit volume of DNA at doses below 50 MGy with low *LET* photon radiation (black curve, cf [7, 12, 13]). This is not the case for light ions where the wide bushy δ-electron (28) track ends may increase the probability for dual local events and more, as seen by their higher event probability at low fragment lengths (red and violet N and He curves seen in the present figure)! The figure is modified from (7, 9, 34) with permission [cf [7].: Figure 65c].

**Figure 7:**
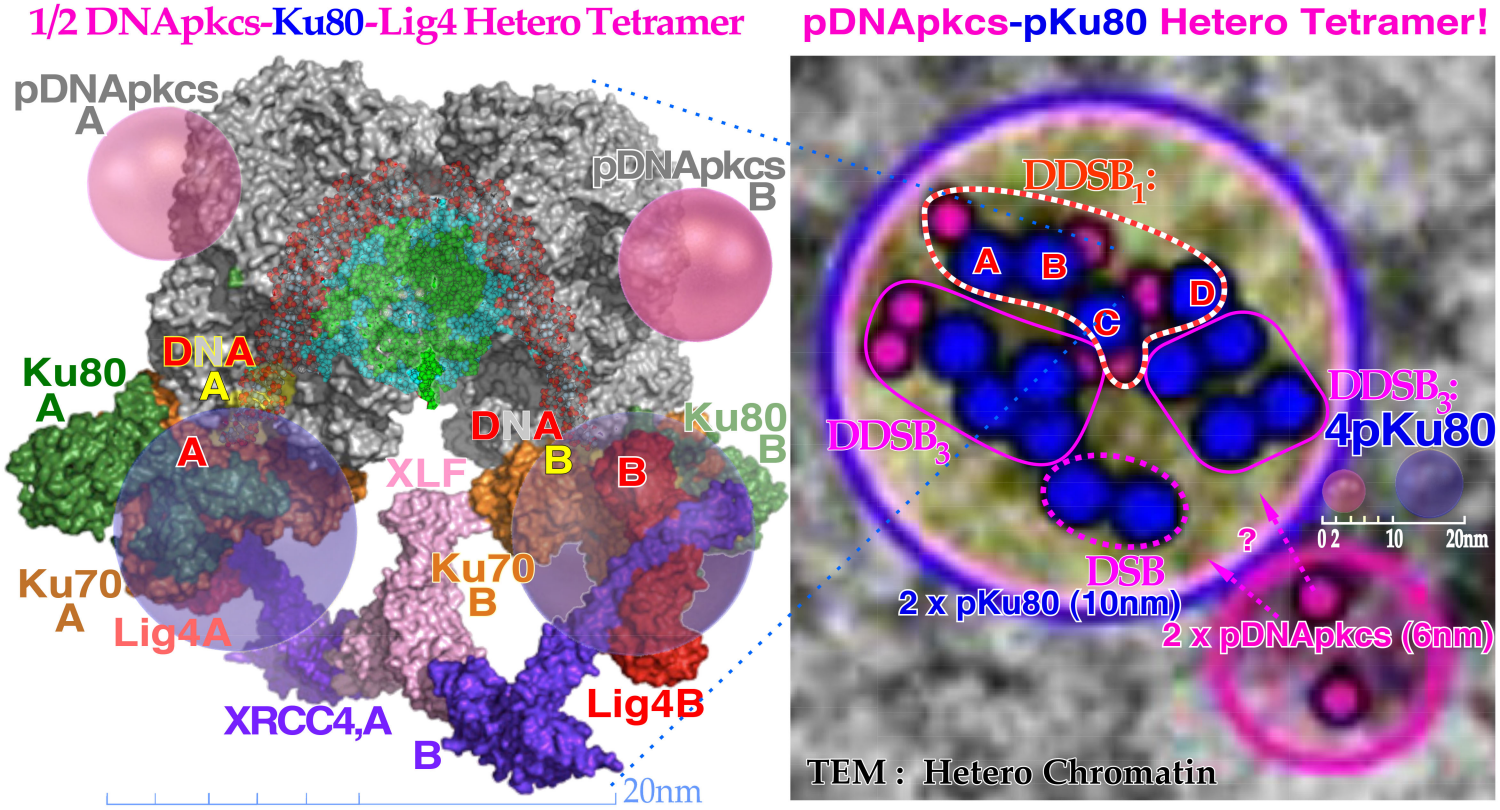
Left: Molecular view of a **δ**-electron track end produced dual DSB at the periphery of a nucleosome and the A and D breaks are connected by a ≈35-nm or a ≈78-base pair DNA strand as seen in **Figures 6** and **8** and (9): Figures 60 and 61, whereas the B and C breaks continue to surround DNA (9, 15). The partially translucent DApk-Ku dimer complex (modified from (47) 7NFC) working in front of the anterior AB–DSB part of the DDSB at the periphery of a partially opened nucleosome semi-translucently visible through the DNApk trying to repair the damaged DNA ends A and B back together by recruiting the lower parts of the DNApk-Lig4 dimer complex with its associated big bluish Ku80 nanoparticles. Right: A close-up view of a dense heterochromatin (dark gray) clusters with seven DSBs and three most likely in the form of DDSBs. A single δ**-**electron may have produced them all. Interestingly, the DDSB with 4× pKu80 (dark blue labeled A,B,C,D) and 4× pDNApkcs (smaller 6-nm dark pink beads inside black circles) is seen inside the red dashed curve in the upper DDSB cluster almost as expected from 2× the left ½ DDSB_1_ (9, 15). The figure is modified from [ (9): Figure 64b, (15),].

At least 80% of the local-energy deposition of all high-energy particle beams such as electrons, photons, neutrons, and ions is deposited by secondary electrons. The very high energy deposition density by the resulting low-energy secondary-electron slowing-down spectrum, in the region of a keV down to approximately 100 eV, is especially severe in terms of their radiation biological effects as clearly seen in **Figures 2**–**4**. Considering their high local doses and the effective *LET* (≈50 eV/nm [1, 3, 5, 7, 8, 12], cf. also upper right corner of Figure 4) of these *δ*-electrons, it is not surprising that these low-energy electrons will have a relative biological effectiveness (RBE) of around 3, as indirectly seen in **Figures 2** and **3**. High-energy photons and electrons above a few hundred keV have a reduced fluence per unit dose of low-energy *δ*-electrons, and their RBE is, by definition, = 1, whereas low-energy ions mainly generate low-keV *δ*-electrons and obtain an effective RBE of approximately 3 at their Bragg peaks as seen in **Figures 3** and **4**. Interestingly, the underlying physical interaction mechanism behind the high RBE of the δ −electrons as well as “twice” for the light ion is that they both are due to velocity resonances with the orbital electrons in the body tissues. Because they have similar speed, they can travel long distances together and therefore get a chance to transfer lots of extra energy as seen in **Figures 2**-**4**, and the resulting probability of producing DDSBs is very high (cf. also Figures 5–7).

**Figure 8:**
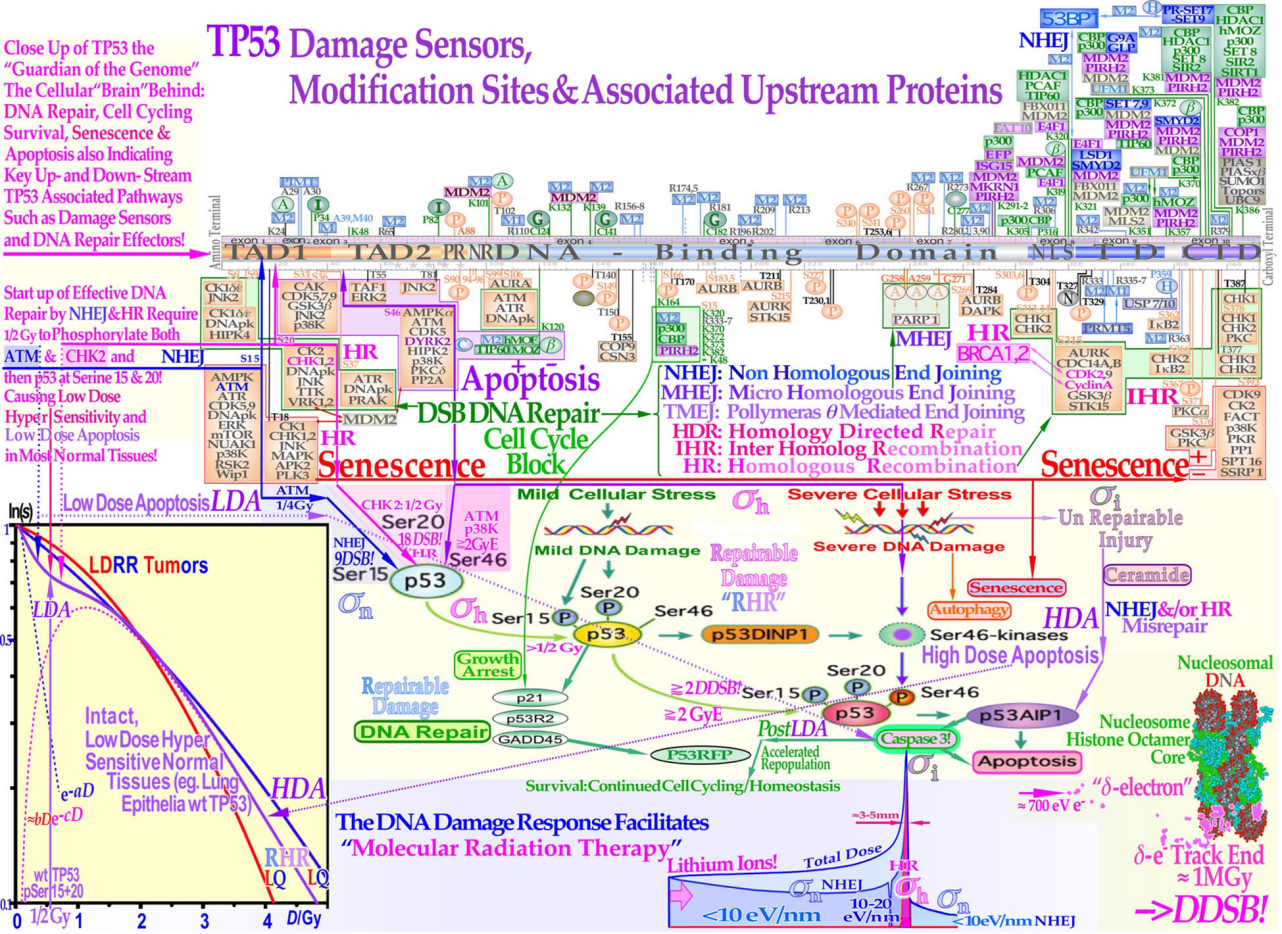
TP53 largely determines the cellular response to different types of DNA damage such as radiation beams (1, 7–10, 12–15). Mild stress phosphorylates the serine 15 and 20 sites on p53 by ATM and CHK2, resulting in cell cycle block and DNA repair. This results in LDHS in normal tissues but generally not in tumors that often suffer from a mutant TP53 gene, as seen in the cell survival insert [cf. also Figures 9 and 18]. Local high doses and high ionization densities (*LET*) result in DDSBs (dual double-strand breaks as seen in the lower right corner (7, 9, 10, 15, 16)) that increase the severity of the damage as seen in **Figure 6**. Thus, it is capable of phosphorylating the serine 46 site, e.g., via p38K or ATM, and a high-dose apoptotic (HDA) response may get triggered (cf Figures 9 and 18). DDSBs are the most common multiply damaged site, and their probabilities determine the biological effectiveness of different types of radiation (cf Figures 2–7) (9): section 6. The lower panel indicates that helium and lithium–boron ions allow a unique therapeutic use by inducing massive apoptotic-senescent tumor cell response mainly within the Bragg peak (σh, σi: cf [1, 7].: Figures 26 and 27). In front of and beyond the Bragg peak, the *LET* is low, and nonhomologically fast and easily repairable damage is mainly induced (σn (7, 9):). This unique property of the lightest ions can be best characterized as allowing molecular radiation therapy since the highest possible apoptosis and senescence can mainly be induced in a 5-mm size spot with mainly low dose and especially low *LET* in the surrounding normal tissues with a predominantly low level of milder type of DNA damage (1, 7, 9, 10, 15, 63). (Figure 1 for further downstream pathway details and more recent on miRNA influences (56, 74)). The figure is modified from (1, 7: Figure 47) with permission.

To really understand the full clinical significance of the sub-keV δ -rays, we need to look at what happens in electron beams as shown in **Figure 2**. The classical plot of high-energy electrons by Berger from ICRU report 35 (28) shows the continuous shift in curve shape as the scattering power increases with decreasing energy. As the electrons penetrate deeper in water, their scattering angles increase and so does the absorbed dose as they travel longer pathlengths at a certain depth interval and thus their practical range is also reduced in units of r_0,_ but their scaled lateral transport is very similar. This continues more and more and when the energy drops below ≈2 keV, a drastic change takes place as seen to the left in **Figure 2** due to the low-energy δ −electron interactions. For 100-eV δ −electrons, the scaled energy deposition distributions are increased ≈20-fold mainly due to multiple Coulomb scattering, multiple scattering detours (29), secondary electron production, and their intrinsic peak *LET* (≈25 eV/nm [2]: Figure 18 [1, 3, 7, 8, 24]), but due to these processes, their effective *LET* reaches ≈50 eV/nm and more as seen in the upper right corner of **Figure 4** (1, 3, 7, 9, 24, 28, 30–33). A recent analysis based on nanometer resolution electron microscopic imaging of DNA damage, based on gold nanoparticle tracers of phosphorylated Ku70/80, DNApkcs, and 53BP1, has identified the key effector of curative radiation therapy for both carbon ions, electrons, and photons to be the DDSBs at the periphery of nucleosomes [DDSBs (9, 15, 16)]. With this method, the DDSBs are clearly visualized as four closely located pKu70 or pKu80 sites in specific configurations, and they are most effectively generated by sub-keV δ -rays [cf. Figure 5 (7, 12)] as proposed almost 30 years ago (9, 16: section 7, Figures 58–61). Ordinary DSBs are also seen as tight pairs of pKu70/80 sites, but as clearly shown in **Figure 5**, more than 99% of these plain DSBs are effectively repaired within a few hours (cf [7]: Figures 51, 83)! Interestingly, the few δ -electrons from 100 eV to 1 keV as shown in **Figure 2** are the real effectors of lethal cell kill and there are between 0 and 4 of them per cell nucleus at 2 Gy as seen in **Figure 5** (on average, 1.5 (7): Figure 15 (12)). According to **Figure 5**, the mean lethal hit number is ≈0.69; thus, on average, ≈2.2 δ -rays are needed to induce a low-*LET*-induced DDSB-based cell kill (Figure 3 as derived in (7): Figures 16, 55–59), truly making the DDSBs and δ -electrons the key effectors of curative radiation therapy (1, 9, 15, 16)! Thus, the scientific question is now: how to deliver these δ-rays safely only to the tumor cells? This is partly what this paper and the book (9) is about. One answer is very simple: Use advanced molecular radiation biologically optimized inversely planned electron, photon, and light ion radiation therapy as seen in **Figure 1**! The most unique and efficient method will be molecular radiation therapy with lithium ions, as they combine low ionization density with easily repairable damage everywhere in normal tissues except in the tumor, whereas the highest possible apoptosis and senescence (≈50% more than carbon ions (9): Figure 102) is induced in a ≈5-mm-diameter volume around the Bragg peak (cf Figure 8) (3, 7, 9, 10). Thus, they are largely saving the unique and important classical daily fractionation window characteristics of low *LET* electron and photon beam therapy (1, 9, 10, 15, 16). However, many other fundamental phenomena are involved on the way, not least using the quantum biology optimization of curative radiation therapy, as recently reviewed (1, 9, 15, 16)! The variation of the RBE with the ionization density (*LET*) of different ions and equally by their mean δ-ray multiplicity along the track is shown in **Figure 4** (9). The dashed curves in **Figure 4** are taken from (24) and describe the average response of the multiple experimental data sets (modified from (25)) very well as the δ-electron multiplicity increases along the ion tracks (1, 9, 15). The ion RBE peak at approximately an *LET* of 120–200 eV/nm thus corresponds to an average *δ-*electron track end multiplicity along the ion track of ≈3 and higher and consequently an RBE ≈ 3. Radiation therapy beyond an RBE of 3 is too extreme with plenty of unnecessary overkill (cf [7].: Figures 62–65 [12]), rapidly reducing the therapeutic effect as seen in **Figures 3** and **4**.

A single or a couple of DDSBs are generally sufficient (15), often making boron ions a better choice than carbon (cf [7].: Figures 94, 97, 98, and 106 [26, 22])! Interestingly, the imparted energy by DDSBs exerts a cell damage event, which is equal for high and low *LET* since all sparsely ionizing electron, photon, and proton beams also mainly work via δ -electrons, making them a fairly common cell kill event even if they are ≈3-fold more common in high *LET* beams. Furthermore, radiation types with a high fluence of such low-energy secondary electrons (24, 26–28, 30–42) are known to have a high RBE as seen in **Figures 3** and **4**. The lightest ions from helium to boron are known to generate high densities of such δ -electrons mainly at the end of their tracks with RBEs in the 2–4 range. Protons also do, but over such a small range (<50 μm) that it is almost totally diluted by their range straggling (≈2 mm, cf [7]: Figure 90). Therefore, the average *RBE* in a proton beam is only around 1.1. Also, low energy photons and electrons in the low-keV energy range have the same elevated *RBE* property but are difficult to use clinically (except possibly through Auger emitters) due to their miniscule penetration in tissues. Light ion paths are aligned by a dense core of δ -electrons, with a surrounding penumbra region of higher-energy δ -electrons characterizing the maximum hit range of an ion but with lesser biological significance (cf. Figures 2–4, and [7]: Figures 14, 23, and 63).

## 3 Dual nucleosomal double-strand breaks are key effectors of curative radiation therapy

Double-strand breaks (DSBs) on the DNA of the cell nucleus are often assumed to be the principal lethal event for the cell. Today, we know that at normal therapeutic doses of radiation therapy (approximately 2 Gy) and ionization densities below ≈100 eV/nm, approximately 75 DSBs are generated in each cell nucleus (cf [2].: Figures 55 and 83). However, less than 1% of them are on average lethal (≈0.9% (9, 10, 15, 16)), at least for sparsely ionizing electrons and photons (and ≈ protons) in normal tissues as illustrated in **Figure 5**. At higher ionization densities, the number of DSBs increases partly due to the more concentrated ionizations along the ion track. However, the number of DSB per repairosome also increases as more and more local DSBs can be handled by the same repairosome ( (3): **Figure 1** inserted). One may therefore ask: what is then really killing the cells? The answer has been known for quite some time, and it is linked to more complex damage such as multiply damaged sites (43). The most common such event is the DDSBs at the periphery of a nucleosome as seen in **Figure 6** (9, 10, 15, 16), cf. also [2]: Figures 26 and 27.

**Figure 9:**
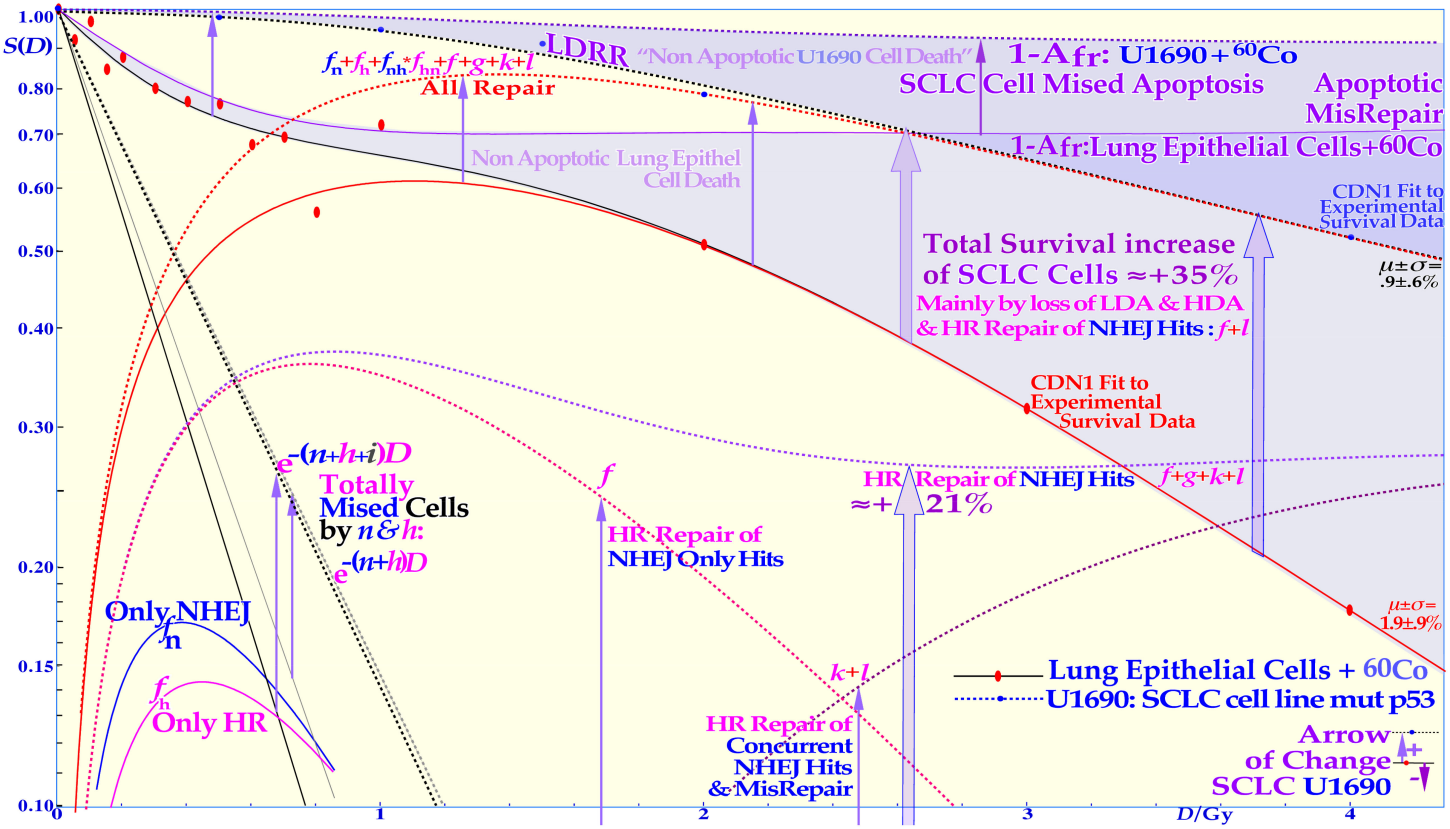
The normal lung epithelia (solid lines) compared with a small cell lung cancer (SCLC) cell line showing a substantially increased survival due to its LDRR phenotype likely to be caused by its TP53 mutations (1, 7, 9, 65) as known from the 1990s. It is seen that practically all the LDA is lost and most of the HDA too without a functioning TP53 pathway. The SCLC cells were irradiated without hypoxia; thus, in a clinical hypoxic tumor, its response may look even worse (cf [7]: Figures 126 and 127). The normalizing effect of reactivating this TP53 mutant cell line by PRIMA 1 has been shown to make some apoptotic normalization (+15%) and simultaneously improving the therapeutic effect of reactive oxygen species (ROS,+27% at 4 Gy (7): Figures 41, 120 and 121, cf [60, 61]). The figure is modified from (9): Figure 40 with permission.

**Figure 10:**
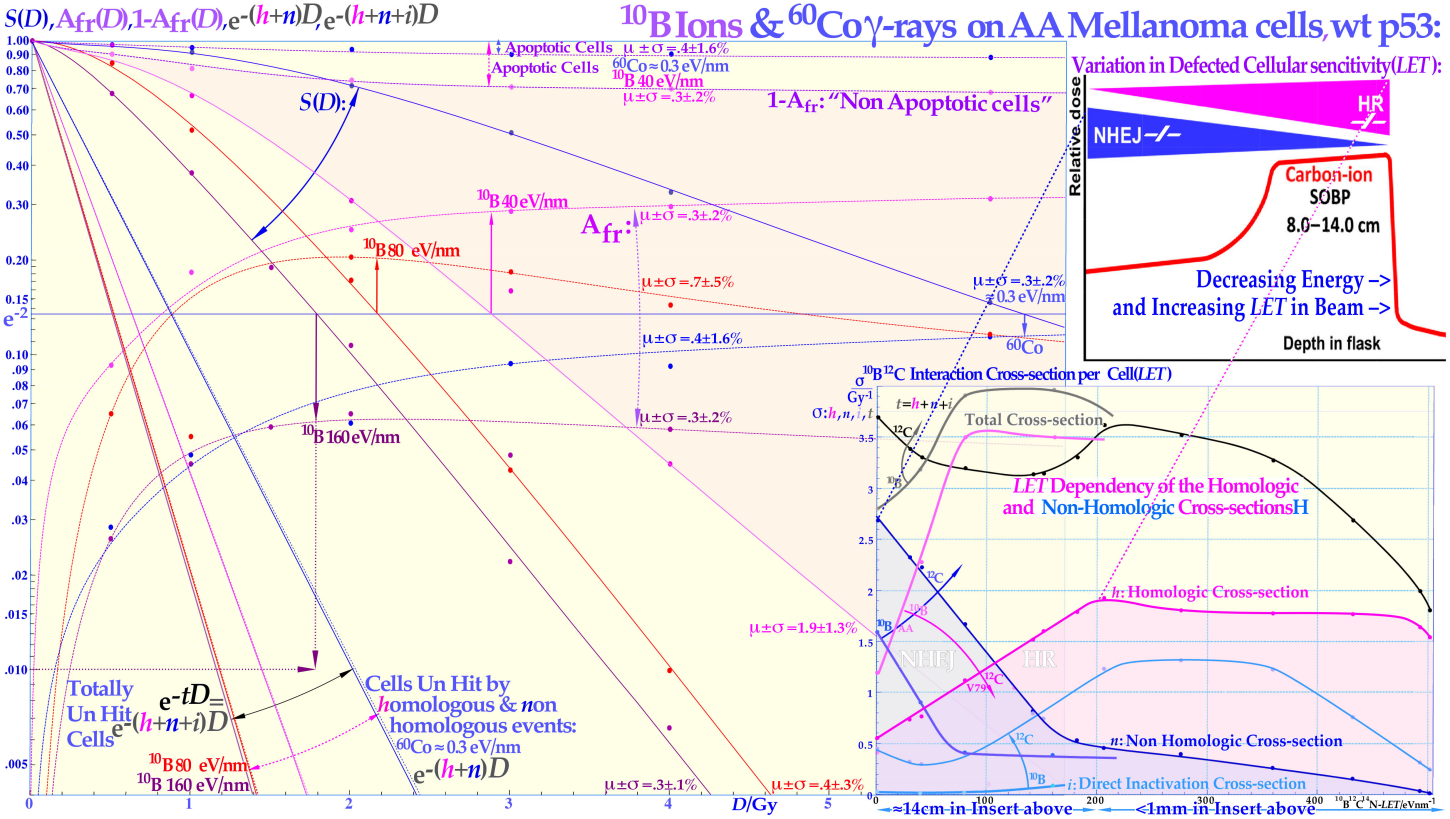
The cell survival, the cell fractions that are totally unhit by the beams, and the apoptotic and non-apoptotic death over the *LET* range 0.3–40–80–160 eV/nm by ^60^Co to boron ions. The cell survival shows a gradual increase in steepness with increasing *LET* whereas the A_fr_ has maximum at a dose causing approximately 13.5% cell survival as indicated by the arrows. For the two lowest *LET*s, the non-apoptotic cells, upper dashed curves, and the clonogenic survival are practically tangential at low doses, indicating that apoptosis is the preferred way of cell death before p53 is phosphorylated at its serine 15 and 20 sites at ≈½ Gy. The shaded area is due to non-apoptotic cell death for 40 eV/nm boron ions. The lower insert shows the *LET* variation of the non-homological and homological interaction cross-sections *n* and *h* for DNA repair after ^10^B irradiation but also ^12^C ion data [as determined in (10): using Eq. (34a)]. The homological cross-section *h* increases very fast with the *LET* for ^10^B ions due to rapidly narrowing *δ-electron cores and the associated reduction of the n* cross-sections. The recent carbon ion insert (upper modified from [66 see also 67, 68]) shows that the change in sensitivity of NHEJ- and HR-deficient cell lines as a function of depth (and thus *LET*) is in total agreement with the *n* and *h*^12^C cross-sections (follow the fine dashed lines), 5 years previously derived in the lower insert now with similar pink and blue shading to clearly demonstrate the agreement (cf [1].: Figure 8 [14]: Figure 18)! The figure is modified from (1, 3, 7, 9) with permission [cf. 7: Figure 45, 15: Figure 18].

Compared to DSBs, the much more severe DDSBs at the periphery of nucleosomes are truly the most common multiply damaged site (43), as seen in **Figure 7**, and (9): Figures 56–60, and are much more lethal (1, 7, 10, 15, 34–42). In fact, the mean cellular potential lethal hit number of 0.69 at 2 Gy is most likely related to the mean ≈1.5 δ-electron track ends (cf. Figure 5) produced in the cell nucleus on average at this dose level and indicates that, on average, approximately half of them may hit nucleosomal DNA (43). A widening of the principal DDSB peak is also seen in **Figure 7** for the nitrogen and helium fragmentation curves relative to photons that are always produced by a single δ-electron. This really explains that the DDSBs are the most common therapeutic entity of low and high *LET* radiations (9, 15, 16), as summarized in **Table 1**. The figures near the small peaks indicate the approximate relative volume of DNA at risk for the different types of δ-electron hits as indicated by their dual arrows of damage (mainly in the straight paths between the pair of dinucleosomes). It is again slightly sharper with photons that totally lack δ-electron multiplicity, making the dual radiation action theory misguided below 10-MGy doses (9, 16)! However, practically both the dinucleosome volumes contribute to the single large DDSB peak. By comparing the red nitrogen curves at 80 and 200 Gy, it is seen that when they are normalized at the DDSB peak height, the absorbed dose dependency is largely eliminated [7: Figures 58–61] and the small random variations may partly be due to the ≈5% uncertainties in the background subtraction process. Clearly, the individual nucleosomes are the key structural component of DNA damage shown in further detail in (9). The tetra-nucleosomal contributions are largely removed by their rather high background at ≈610 bp (9): Figure 65b (15, 16). Interestingly, this key effector of cell death, the secondary δ-electron track ends of between 1.2 and 0.2 keV, can deliver ≈0.3–1 MGy to hundred nm^3^ size volumes in the cell nucleus (cf. Figure 4). This is mainly due to multiple Coulomb scattering (9): Figure 19, multiple scatter detours (29), secondary electron production, and their intrinsic low to medium *LET* (≈10–25 eV/nm (26)), but their effective *LET* reaches ≈50–60 eV/nm and more as shown in **Figure 4** (cf [7].: Figures 18 and 19). At the common low *LET* therapeutic dose of ≈2 Gy, there is only 1–2 such track ends per cell nucleus as shown in **Figure 7**. Importantly, this is also the key effector of therapeutic light ion beams. Due to their higher secondary δ-electron production (8, 9, 28, 30–32, 34–42) of approximately three to five per cell nucleus at 2 Gy, they are approximately three times more toxic than plain high-energy photon and electron beams (cf [2]: Figure 15). This is truly the main reason for their higher RBE, even though they too only make ≈75 DSBs just like electrons and photons, at that dose level as seen in **Figure 5**. However, now approximately three are DDSBs (≈6 DSBs); thus, ≈70 are ordinary plain DSBs. To the right in **Figure 8**, a nanometer resolution view of a DDSB imaged using 6- and 10-nm gold nanoparticles indicates pDNApkcs and pKu80, respectively. Interestingly, the molecular view to the left describes half the DDSB (anterior part of the nucleosome) quite well, now enlarged approximately four times! The repair problem arises as the A and B ends cannot be brought together without partial dismantling the nucleosome at the same time as D on the posterior half of the DDSB is connected to A by a ≈35-nm DNA string (cf. Figure 6). This will most likely force the normal second Ku-DNApk dimer complex needing to be replaced by Rad50s coiled coils with higher geometrical flexibility (≈100 nm) using the MRN dimer complex of the HR repair system for correct repair if at all possible as discussed in more detail in (9, 15, 16, 27, 48–57). The upper right DDSB in ≈nm resolved heterochromatin is in good agreement with the left molecular view of a full pDNApkcs-pKu80 tetramer (only one-half is shown to the left with the anterior DSB) at approximately three to four times larger scale even if the DDSB is far from repaired yet. At this stage, it is often likely that the last posterior DSB pDNApkcs-pKu80 dimer is being replaced by the MRN dimer complex of HR to use the Rad50 coiled coil flexibility to try to finalize the repair (cf. Figure 10 right inserts and [7]: Figures 60 and 61 [8, 47])!

**Table 1:** Summary of the main reasons why δ-ray-generated DDSBs are the key effectors of radiation therapy.

1) More than 80% of the energy deposition by different radiations go via δ-ray DDSB (cf. Figures 2–4 [7]).
2) The DDSB is the most common multiply damaged site generating severe damage to DNA (cf [7]).
3) In contrast, plain DSBs are easily repairable by NHEJ and HR; more than 99% are repaired at 2 Gy (**Figures 5**, **7**).
4) The δ-ray RBE is high (≈3) with a high probability of generating DDSBs (cf. Figure 2).
5) The ion beam RBE is closely equal to the mean δ-ray multiplicity along the track (cf. Figure 4).
6) The DDSBs are practically ≈unrepairable by the NHEJ repair system based on DNApk (cf [12, 13]).
7) The δ-ray DDSB generating local dose is very high ≈1 MGy, causing severe local damage (cf. Figure 4).
8) There are very few DDSBs<2 Gy due to the low *LET* normal tissue fractionation window (**Figure 13**).
9) The electron-specific energy is >2 below<1 keV of energy (cf. Figure 2).
10) Approximately 40% of all ion DSBs are DDSBs (cf [2, 8]).
11) DNA low length fragmentation is dominated by DDSBs and is very similar for high and low *LET* (**Figure 6**).
12) As the δ-ray multiplicity along the track goes up, shorter fragments are generated by higher *LET* ions (**Figure 6**).
13) The electron proximity function has its maximum at 2 nm (cf [7])!
14) Ions near the Bragg peak have an RBE of approximately 3 as the δ-ray multiplicity along the track is ≈3 (cf. Figure 4).
15) When NHEJ get stuck on a DDSB, homologous recombination may take over via the MRN complex (cf [7, 12, 25]).

## 4 Radiation biology of TP53 in tumor and normal tissue cell survival

**Figure 11:**
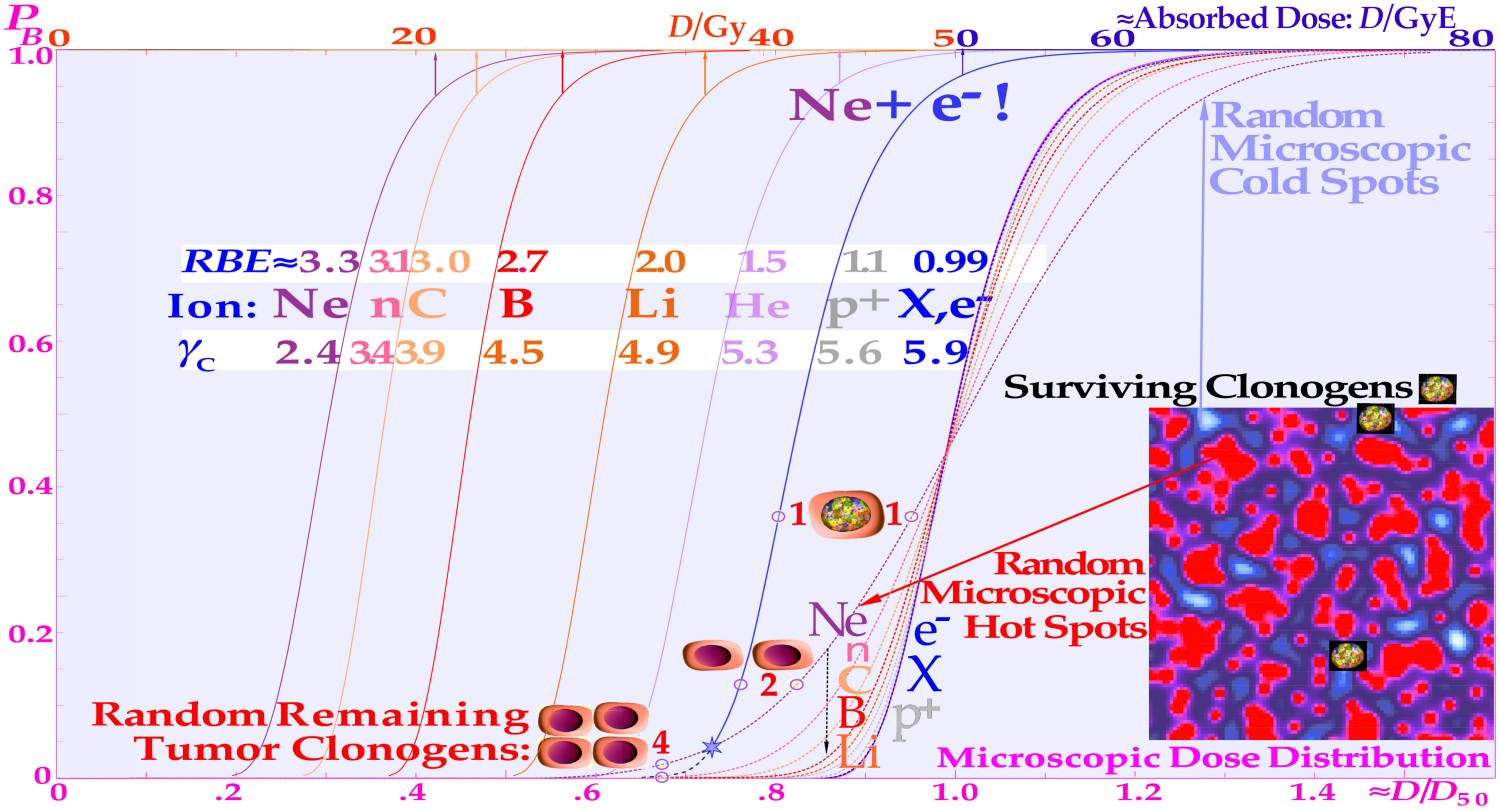
The tumor control probability curve for a uniform cell line using different radiation modalities as a function of the absorbed dose (upper scale). The lower scale and dashed curves are normalized to the ≈50% tumor control dose that is approximately proportional to the dose equivalent, to more clearly show the effect of the microdosimetric relative standard deviation *σμ on the γ***_C_** dose response slope by increasing the *LET* (cf. digital values in the table). Not only are the hot spots often in the form of DDSBs (cf. Figures 5 and the lower right corner of 8 (7), (25); and cold regions become more extreme with increasing *LET*. The RBE first increases, thus reducing the total dose by approximately threefold with carbon, neutrons, and neon, increasing the relative standard deviation, and reducing the *γ*c value more than therapeutically desirable. For a real low-*LET* roundup treatment such as the schematic neon ions+electrons, an extra upper Gy-Equivalent scale is needed, indicating a substantial dose equivalent gain of ≈15 GyE, and about half that value for carbon ions+electrons. The figure is modified from (1, 9) with permission [cf. 7: Figure 74].

**Figure 12:**
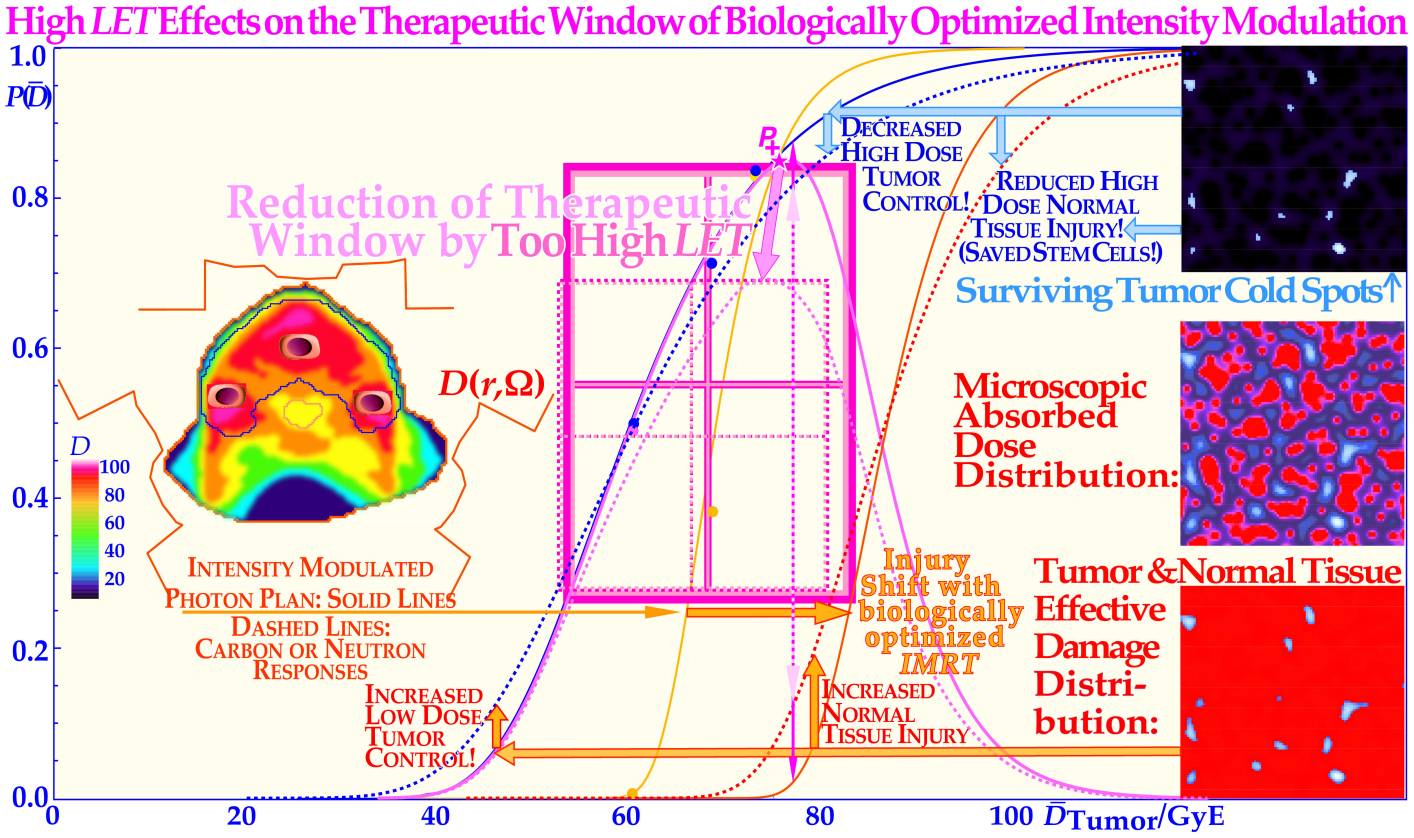
A very high *LET* (>5 eV/nm) has adverse effects on the complication-free cure (*P*_+_) as it reduces the high-dose tumor cure and simultaneously increases lower-dose normal tissue injury [dashed lines; solid lines from (70):: **Figures 1** and **7** (69, 71, 72)]. Interestingly, there is a very cost-efficient clinical solution to this problem: by switching to electrons, photons, and, in special cases, even protons during the last week of treatment. This will lead to a steeper tumor response as seen in **Figure 11** [(5): Figures 20 and 22 (7): Figures 74, 79, 106, and 112 (25): Figures 8.10 a, b (53, 60, 72)], generating a higher complication-free cure. All delivered at a lower total dose as also shown in **Figure 11**, with the consequence that the high-dose tumor cure is improved and the normal tissue damage is decreased. Taken together, this results in a significant increase in the complication-free cure and the width of the therapeutic widow as clearly seen in this figure. *P*( $\bar{D}$): the probability of tumor control or normal tissue damage as a function of the mean tumor dose. The figure is modified from (1, 9) with permission [cf. 2: Figure 82].

The recently understood differences between tumor and normal tissue responses derived using the repairable-homologous-repairable (RHR) DNA damage repair formulation (7, 9, 10, 15) require renewed thinking about many aspects of the biological optimization of radiation therapy. It has been suggested that most TP53-intact normal tissues are generally low-dose hypersensitive (LDHS, see the lower left insert of **Figure 8** and **Figures 9**, **10** (1, 9, 10, 12–15)) and that the inherent microscopic heterogeneity of higher *LET* ion treatments in the last week of treatment would benefit substantially from a low *LET* roundup, (cf Figures 11 and 12) (1, 7, 9, 12–15). The quantification of apoptosis (7) has helped identify the early low-dose hypersensitivity and low-dose apoptosis of most normal tissues and tumors with intact TP53 and ATM genes (1, 7–9, 12–15). This mechanism has probably been developed early on by nature’s natural proses of preferential survival advantage selection, to ensure minimal risk for severe mutations to the genome, before the DNA repair system is fully functional after a dose of >½ Gy (1, 7–10, 12–15). The need for this mechanism was already pointed out in (9): Figure 19, where it was shown that if one DNA strand was hit by a *δ*-electron, the probability that also the other strand may get damaged was maximal due to the intrinsic property of the so-called proximity function associated with the scattering properties of low-energy δ -electrons below ≈1 keV as this function has a maximum at distance of ≈2 nm (9): Figure 19.

**Figure 13:**
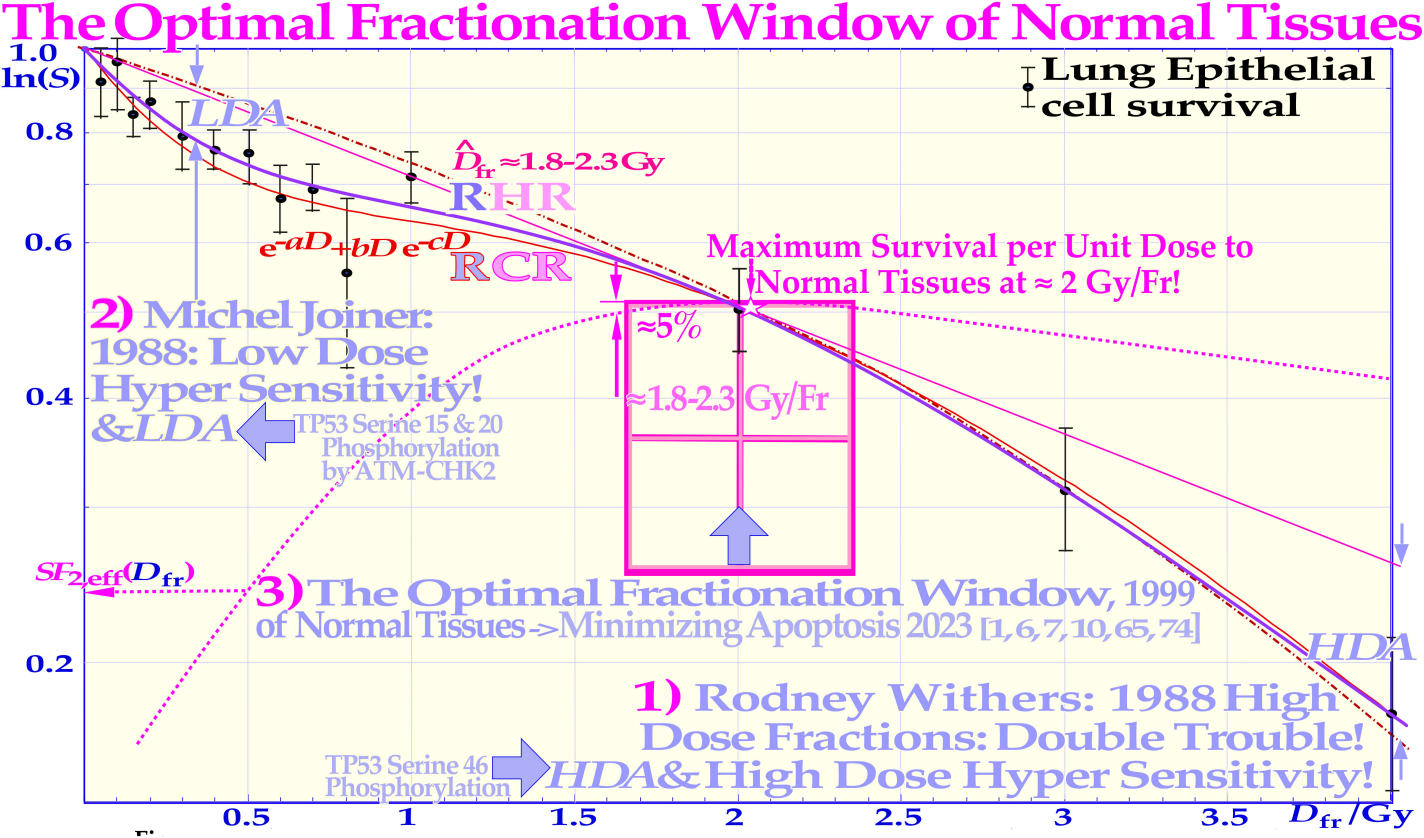
The fast low-dose initiation of full DNA repair first causes low-dose hypersensitivity (LDHS) and low-dose apoptosis (LDA), but it is followed by almost a plateau of effective repair and improved cell survival per unit dose. In normal tissues at risk, there is therefore generally a daily fractionation window ≈ 2 Gy/Fr (≈1.8–2.3 Gy cf [7].: Figures 50 and 81 [7]), where the least detrimental response is obtained (*SF*2 ≈ 0.52 and *D*0,eff ≈ 3.1 Gy) for a given therapeutic dose level having to be delivered to the neighboring tumor volume (cf [7].: Figures 49 and 50). The magenta dotted curve shows how the effective surviving fraction at 2 Gy (*SF*2,eff) varies with the dose per fraction on the horizontal axis, having a clear maximum near 2 Gy. This is probably the main reason why, in classical radiation therapy, the tumor and normal tissue doses were often rather similar due to parallel opposed beams and no use of IMRT, and the dose delivery was best tolerated ≈ 2 Gy per fraction. The figure is modified from (9, 71) with permission [cf. 7: Figure 78].

**Figure 14:**
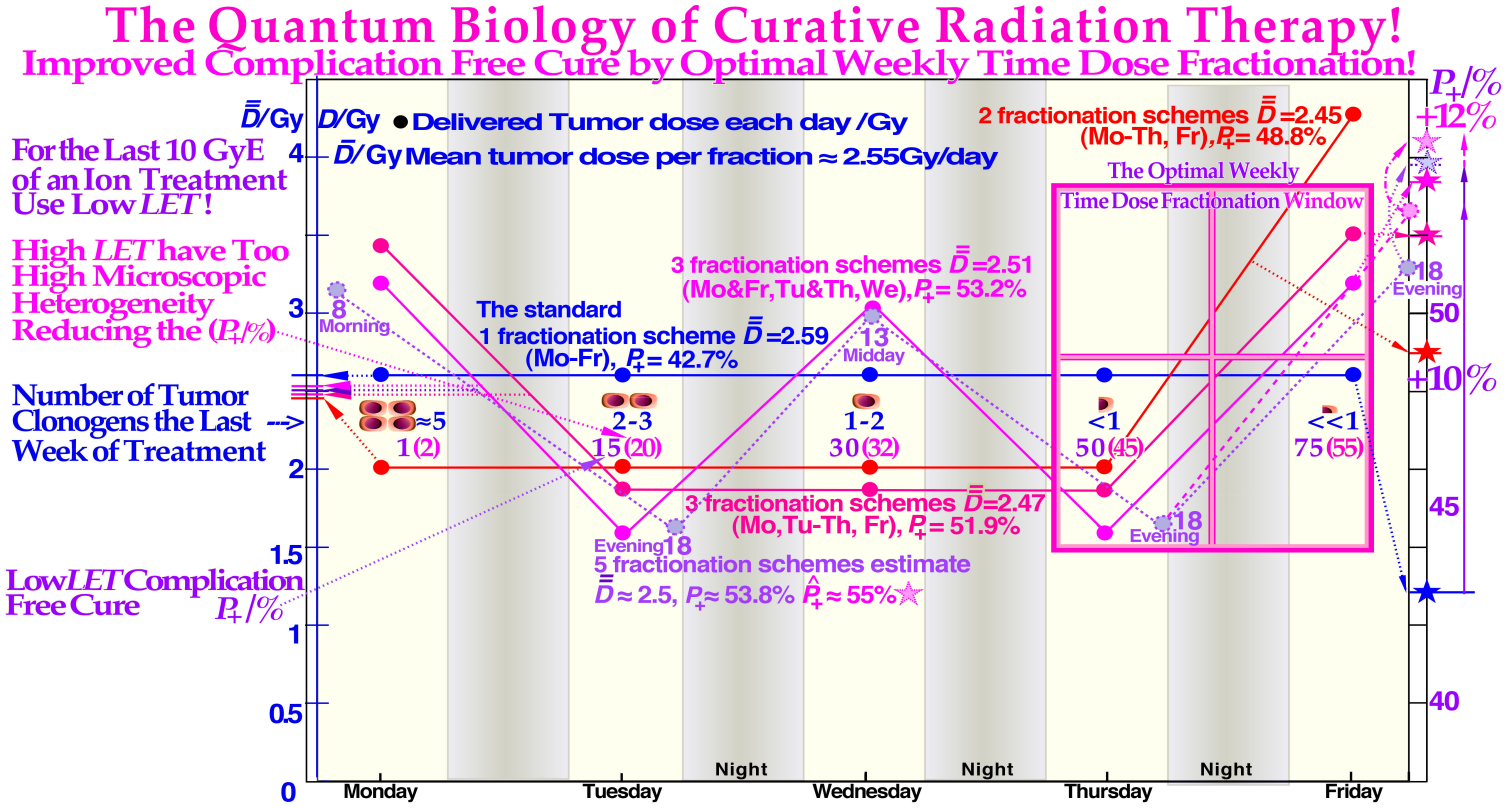
Using the optimal weekly fractionation schedule, an improvement in complication-free cure by up to 12% (*P*+, right scale) is seen, making the weekend free from treatments and maximizing the sublethal damage HR repair in normal tissues as seen in the lower right insert. Interestingly, the improved normal tissue repair capability is particularly valuable during the last week of treatment when the tumor burden is considerably reduced and normal tissues suffer the most after several weeks of treatment. Nevertheless, the last treatment fraction should be high to use the extra repair potential at the end of treatment. Clearly, the advantage of a low-*LET* roundup after a high-*LET* treatment will generally optimize the whole treatment procedure, not least using the present fractionation approaches. As seen in the middle row of cells and violet numbers, a low *LET* roundup can regain the highest possible *P***_+_**and complication-free cure. The figure is modified from (3, 9) with permission [cf (7).: Figure 84 and (79)].

As a compensating measure for the induced apoptosis, the apoptosis-inducing caspase 3 gene product (**Figure 8**, lower right) remarkably “remembers” this protectively induced LDA cell loss and starts accelerating cellular repopulation to reestablish homeostasis in the tissues after being irradiated. With a very high *LET*, apoptosis and senescence will instead be high in the normal tissues in front of and behind the tumor, which definitely is undesirable from a complication-free cure point of view, even if hypoxic tumors sometimes may marginally benefit from a higher *LET* (cf [7].: Figures 112, 114, and 117 [12]). It is well known that the nonhomologous end-joining (NHEJ) pathway, (cf. Figure 10 inserts (1, 3) is the dominating DNA repair process at low *LET*, and it is very fast. Ku70, Ku80, and a DNApk dimer bind together the broken DNA ends in a few seconds and simultaneously recruit p53 (12, 48, 50), such that the multiple DNA strand ends at high-dose and *LET* local damage can be repaired together in the right order except possibly for the *δ-*electron-generated DDSB produced in the same ≈picosecond (15). The effect of high dose rates is discussed in further detail in the book (9) in Figure 67b.

This process is essential especially at high *LET* levels when the MRN dimer complex often replaces Ku-DNApk heterodimers not least if the cell is in the S or G2 phase of the cell cycle, and the homology-searching mechanism and higher flexibility of homologous recombination (HR) are needed for high-fidelity repair (15, 48, 57). The new consideration of HR repair of NHEJ mis- repair makes it possible to describe cellular repair far beyond the conventional linear quadratic model (LQ). Interestingly, the new DNA repair-based formulation inherently describes LDHS and LDA as they are linked to the DNA repair system of most, if not all, normal tissues, as described in more detail in **Figure 8** (7, 9, 15). This figure illustrates how the TP53 gene works as a complex cellular mastermind and controller by determining how depending on the structure of DNA damage it should best be repaired and whether senescence and apoptosis are needed (3, 7, 9, 10, 48, 56). As seen from the lower left insert in **Figure 8** (cf. also Figures 13 and 18 and [2]: Figures 48–50), after a total of ½ Gy or 18 DSBs, CHK2 is also phosphorylated and so is the serine 20 site on p53, which results in a gradual switch in normal tissue sensitivity from an initial LDHS stage to a more radiation-tolerant almost LDRR-like state. In fact, the well-known experimental demonstration that the LDHS property can be eliminated by low-dose preirradiation is a clear indication that the first ≈½ Gy is needed to start up efficient DNA repair! Interestingly, after that, the cellular repair system is fully activated and functional, with reduced cell loss and almost a survival plateau towards 2 Gy (1, 7, 9, 10, 14, 15, 58–62). It is clear from the **Figure 8** insert that doses well above 2 GyE are truly needed for significant tumor cure, but the dose to normal tissues should be just above 2 Gy or close below to ensure optimal radiation recovery (cf also Figures 13 and 18 as is more clearly explained in (9): Figures 49, 50, and 81–84). The LDA and LDHS of normal tissues are caused by 5%–15% acute low-dose apoptosis ( [7]: Figures 47–49 and [1, 4–6]), but interestingly, most likely, owing to the compensating measure of caspase-3-induced cellular repopulation (64), late effects are therefore few as it try to compensate for the LDA apoptotic cell loss. This will re-establish homeostasis in normal tissues and thus minimize late normal tissue damage and furthermore helps generate a fractionation window in normal tissues (cf [7]: sections 5.4–5.7), but it may sometimes also repopulate malignant tumor clonogens if they are not entirely eradicated by the treatment (64). This means that LDA and LDHS truly protect normal tissues from potential low-dose mutations before NHEJ and HR are fully functional and can address the damage (cf [7]: Section 4.7).

Thus, now as we start to understand the magic function and therapeutic property of the 2 Gy/Fr basic low *LET* optimal fractionation window, we can summarize the following:

1. Do not surpass the 2 Gy/Fr Low *LET* dose level to avoid undesirable DDSBs in normal tissues (cf. Figures 4 and 6).
2. Use the acquired radiation resistance and efficient DNA repair in normal tissues induced after 0.5 Gy low *LET* dose as far as possible up to a maximum of ≈2.3 Gy (cf. Figure 7 [7]: Figure 81).
3. Do not surpass the 2.3 Gy/Fr to avoid high-dose apoptosis (HDA) in normal tissues (cf [7].: sections 5.6 and 5.7).
4. Accept the low-dose apoptosis in normal tissues to get “2)” since caspase 3 reestablishes homeostasis by accelerated repopulation when they are no longer irradiated (cf [5–8, 70].).
5. In fact, ≈2 Gy/Fr minimizes normal tissue apoptosis since we have to accept at least 0.5 Gy to be able to treat at all and thus LDA and caspase 3 repopulation. However, HDA and late damage should be avoided (1, 9, 64)!

It is fascinating that, first, after 125 years of curative radiation therapy, we start to understand how the molecular mechanisms behind an intact TP53 gene make 2 Gy/Fr so useful in the clinic. When it is commonly mutated in the tumor, we may need biologically optimized intensity-modulated radiation therapy (IMRT), light ions, and even p53 reactivation, to achieve the best ever complication-free cure of LDRR tumors (9).

## 5 The quantum biology of curative radiation therapy

As seen in **Figures 1** and **11**-**12** when there is only one or a few single viable clonogenic cells left in the tumor during the last week of a curative treatment, it is unsuitable to try to use high *LET* ions to hit them. Even if it is generally our best tool available to treat malignant tumors, we need to know exactly where the clonogens are and where to aim the beam to hit the cell nuclei! In fact, if we know precisely where, e.g., 2*n* cells are located, we need just *n* ions as each ion can hit at least two cells in one shot when we know where they are! If we do not, according to Heisenberg’s wonderful thinking, applied on this simplistic case, we need a beam at least as large as the tumor if we wish to be sure to hit them all [actually a little bit bigger if there are also uncertainties in the beam patient setup (69)] and we need to also consider the quantum biology of curative radiation therapy so we do not get a microscopic ion beam cold spot on some of the clonogens. However, at the beginning of a treatment with millions of hypoxic radiation-resistant tumor cells, ion beams are the most effective treatment since independent of where we aim the ions, we will hit thousands of tumor cells. If you have an ion path through millions of cells, its effect on the tissue is well described by the dose average pencil beam kernel as it is the average response that counts and it is given by taking the average effect on the millions of cells, which is exactly the definition of the mean dose distribution of the beam over cell nuclear sizes! If there are only a few clonogens left, such a mean energy deposition kernel is too crude, and we have to look at the probability that at least one of the remaining clonogens is missed by the beam and may repopulate the tumor (64)! Today, we know very well that this may happen since caspase 3 is likely to step in after the treatment, trying to recover normal tissue homeostasis by accelerated repopulation, which is known to also affect potential remaining tumor clonogens (64).

**Figure 15:**
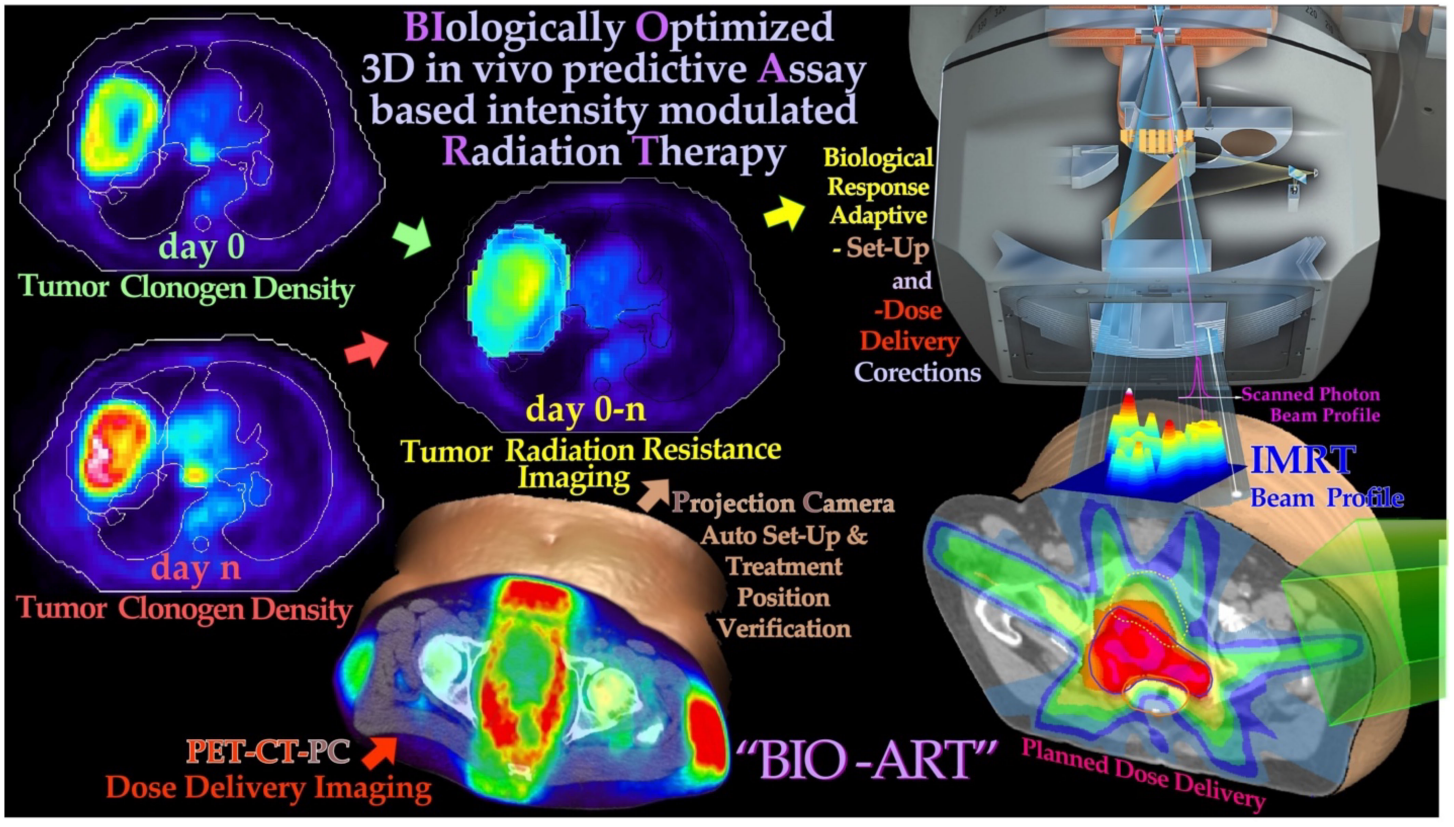
PET-CT-PC can be used to derive tumor responsiveness information that can be used to plan, verify, and biologically optimize using the *in vivo* predictive **a**ssay-based **r**adiation **t**herapy method [BIOART (20, 27, 70)]. By measuring the tumor cell kill early in the treatment, 3D *in vivo* predictive assay information about radiation responsiveness is obtained for accurate prediction of the optimal dose delivery based on measured hypoxic tumor cell response status and adaptive control (4, 11, 27). Different imaging methods from various body regions exemplify aspects of the adaptive feedback procedure (4). The normal tissue responses are approximately known from historical data (20, 27). Both with light ions and scanned high-energy photon beams, 3D *in vivo* PET-CT dose delivery monitoring is possible to further optimize the treatment based on observed mean dose delivery, which may differ from the planned dose delivery without considering organ and patient motion. Both these data sets when used together will allow a high degree of therapy optimization where practically all major sources of treatment error can be picked up as long as they influence tumor cell survival and can thus be corrected for using biologically optimized adaptive treatment techniques [cf [2].: Figure 126, right panel, where the lower part of the tumor was under-dosed by organ motion in and out of the beam with increased cell survival in the lower part of the tumor (11, 23, 27)]. Modified from [7: Figure 125].

Interestingly, if we instead had truly deterministic ion beams where all ions were exactly known to travel perfectly parallel to each other and in a precise hexagonal grid with a separation of, for example, 7 μm and all cell nuclei were perfectly spherical with a diameter of >8.1 μm, all would be hit as the escape radius is 7/√3 ≈ 4.04. In fact, with such a deterministic beam, the mean hit number would be 1.89 but no missed cells (9!) instead of 4.5 at 3 Gy random carbon ions with ≈ 1.2% of missed cells, but astonishingly the microscopically quasi uniform dose is less than 1.3 Gy (<43% of the Poissonian beams we are used to)!!! This means that if we could produce such a beam, we could improve tumor cure and reduce normal tissue damage substantially. Obviously, this could be done by microfabrication of extreme pinhole collimators, but unfortunately, a major part of the ion beam will then be stopped in the collimator and produce a number of decay component lighter ions and neutrons that will be harmful to the patient, even if there exists a well-optimized technique developed for electrons that can be easily modified for ions! Excellent results will certainly be possible using advanced microscopic μm-size pencil beam scanning techniques that have already been developed not only for micro-irradiation of subcellular components in the laboratories (9) but also for electron microscopy. However, they need to be very fast in scanning to avoid extreme treatment times; a 10 by 10 cm^2^ field will have (100,000/7)^2^ beam spots or 200 million spots; thus, a rate of 1,000 × 1,000 has to be achieved in a second for a 3-min treatment time, but we most likely will need 10–20 beam energies, so it will not be easy! Multi-grid ion beam sources may be a way to increase efficiency but will require good beam optics all the way to the patient. It is definitely not a simple task but hopefully worth thinking about! A further problem may arise if we had such a beam as multiple scatter in the patient may allow clonogen misses, e.g., behind bony structures. An interesting possibility would be to produce the ion beam in a dispersion-free accelerator and storage ring with effective electron and laser beam cooling to generate a perfectly “crystalized” ion beam (cf [7]: Figure 70 and [73]). This can produce an almost perfect circular beam consisting of 130 stable ion locations over the perfectly circular beam cross-section. The already proposed low *LET* roundup method is likely to be more straightforward and will surely produce the steepest possible dose responses. Deterministic molecular homogeneous light ion beams will improve responses at ≈58% lower dose but the longitudinal microscopic variance will still remain, which will most likely be a minor problem.

Therefore, like in quantum mechanics, it is generally not possible to state the exact state of the patient after a treatment, whether he or she is cured and alive and well or not; we can only state the probability range to expect, e.g., using the ExtremeValue Distribution (1, 9). This situation could also be compared with the famous enigma of “Schrödinger’s cat” and with Heisenberg’s Uncertainty Principle applied to ion-beam radiation therapy as just discussed above [cf. 7 for details]. In fact, radiation therapy is truly the perfect example of extreme value distribution, as it is well known that only the last few and most likely the most radiation-resistant tumor clonogens have survived the initial major part of the treatment (≈60 GyE/70 GyE ≈ 85%) and not killed. Instead, they remain to finally form the tumor control probability curve, as recently described in great detail ((15): Figure 20). It is therefore not surprising that the tumor control probability can be rewritten to perfectly follow the cumulative extreme value distribution (1, 9) as seen in Equation 1:

(1) $${\text{e}}^{-\text{e}(\mu -D)/v}={\text{e}}^{-\text{e}(D_{0}\text{ln}N_{0}-D)/D_{0}}=\;{\text{e}}^{-N_{0}\text{e}-D/D_{0}},\;$$

where the last part is a rewriting of the Poisson statistical probability for having no surviving viable tumor clonogens at the end of the treatment and therefore the approximate mean value of the extreme value distribution *μ*=*D*_0_lnN_0_ and the “radiation resistance” *v*=*D*_0_ can be identified. To be more exact, the true mean value is actually given by $\bar{D}$=*μ*+v*γ=*D*_0_(lnN_0_+*γ*), the median value is given by *D*_50_=*μ-v*ln(ln2)=*D*_0_ln(N_0_/ln2), the variance is V=*σ*_D_^2^=π^2^*D*_0_^2^/6, and the relative standard deviation is *σ_μ_*=σ_D_/ $\bar{D}$=π/ $(\sqrt{6}$(*μ*/*v*+*γ*))=π/ $(\sqrt{6}$(ln*N*_0_+*γ*)) which, from a microdosimetric point of view, is an important quantity (*γ*=Euler’s Gamma ≈ 0.5772). For a common tumor size of *N*_0_ = 10^7^ clonogens, the relative standard deviation *σ*_D_/ $\bar{D}$≈0.0768, so only approximately 7.7%, thus making the tumor control curve shape quite steep and rather sensitive to microscopic dose fluctuations.

This is partly due to its high Kurtosis = 5.4 independent of μ and ν as well as *N*_0_ and *D*_0_, and to its Skewness ≈ 1.1395 explaining the steeper rise of the tumor control curve at low doses and the shallower extended shoulder at high doses, thus making it generally very hard to achieve 100% perfect tumor cure, as is well-known clinically. The commonly used Gaussian distribution by necessity is linked to zero Skewness and a Kurtosis of 3.0, so it is really unsuitable to describe clinically relevant dose–response relations! Here, we also assumed a perfectly uniform dose delivered to all cells with a fixed radiation resistance *D*_0_. This is not really applicable to ion beams with substantial microdosimetric variance *σ_μ_* as shown in **Figures 4**, **11**, and **12** (1, 9). It is clearly seen that this has a detrimental effect on the clinically observed steepness γ**_C_** of the dose–response relation and the dose equivalent (see, e.g., **Figure 13** for Ne+e^−^) and reduced risk of damaging normal tissues, as demonstrated here. The rather low final dose increments in a high-*LET* and high-microscopic heterogeneity treatment will inevitably generate microscopic cold spots where some of the few remaining tumor clonogens may survive, and it is even more unlikely that the heterogenic hot spots fall on all of the remaining tumor clonogens.

After such a noncurative treatment with caspase-3-induced apoptosis, an accelerated repopulation of normal tissues, as well as of possibly remaining clonogenic tumor cells, may be induced (64).

The risk for severe normal tissue damage is much lower with an electron or photon roundup, and a high *LET* can be fatal with the last few ion fractions where the tumor control truly should increase from a few % to preferably ≈ 95% with practically no remaining tumor cells. This is NOT even possible with high *LET* ions without damaging normal tissues as seen in **Figure 13**! Unfortunately, it seems that many treatments are done today with light ions without a real research approach looking carefully at the resultant complication-free cure probabilities of the treatments! For the lightest ions with largely a low *LET* in normal tissues, such as helium to boron ions, the normal tissue dose should preferably also be in this range unless there is a substantial high *LET* dose spillover to critical normal tissues surrounding the tumor region. With well-optimized IMRT dose delivery, the tumor dose could simultaneously be >1.5 or more times higher, and in addition, the biological effectiveness is significantly increased (cf. Figures 7 and 14). This makes the total increase in therapeutic effect in the tumor approximately two to three times higher and more, especially for smaller tumors.

## 6 Normal tissue sparing by the optimal weekly time dose fractionation schedule

To minimize normal tissue apoptosis and the effect of the LDHS and LDA, there exists an optimal daily fractionation window at approximately 1.8–2.3 Gy/Fr where the shallowest tangent from the origin touches the cell survival curve at approximately 2 Gy (as clearly seen in **Figures 9** and **13** [2, 7, 15]). Using such a dose per treatment fraction in the normal tissues around the tumor also means that the tumor dose is significantly higher, by a factor of approximately >1.5, and the tumor will experience significantly more damage, especially with mutated DNA repair genes (9). In addition to this classical normal tissue daily fractionation window around 2 Gy/Fr (9, 74–77), there is an optimal weekly fractionation window by giving higher doses per fraction on days where there is a longer time for sublethal damage repair before or after the treatment. By this method, it is largely possible to compensate for the two missing dose fractions over the weekend and thereby better maximize HR (and of course NHEJ) repair. The optimal weekly fractionation window is illustrated in full detail in **Figure 14**, showing that simply by increasing the dose on Friday and utilizing the weekend for repair (cf. Figure 14, red curve (right scale and insert), a gain in complication-free cure (*P*+) of approximately 6% can be achieved (**Figures 13**, **14**).

Because of the effective weekend repair, the dose fraction on Monday could also be increased to gain a further 3% in *P* +. A total gain in *P*+ of approximately 12% is possible by giving high doses on Monday morning and Friday evening, lower doses on Tuesday and Thursday evening, and rather high doses midday on Wednesday and the last day of treatment, as demonstrated in **Figure 14** (1, 3, 9, 78). In fact, as shown in **Figures 11** and **14**, this type of schedule will maximize the normal tissue HR repair and minimize the induction of HDA-type apoptosis by accepting the initial LDA after the first ≈ 0.5 Gy (7, 9, 14), simultaneously approximately following the optimal 2 Gy/Fr clinical fractionation window (7, 9)! The LDA is later partly recovered by caspase-3-induced accelerated cellular repopulation of normal tissues (9, 64).

## 7 Biologically optimized in vivo predictive-assay-based treatment planning

**Figure 16:**
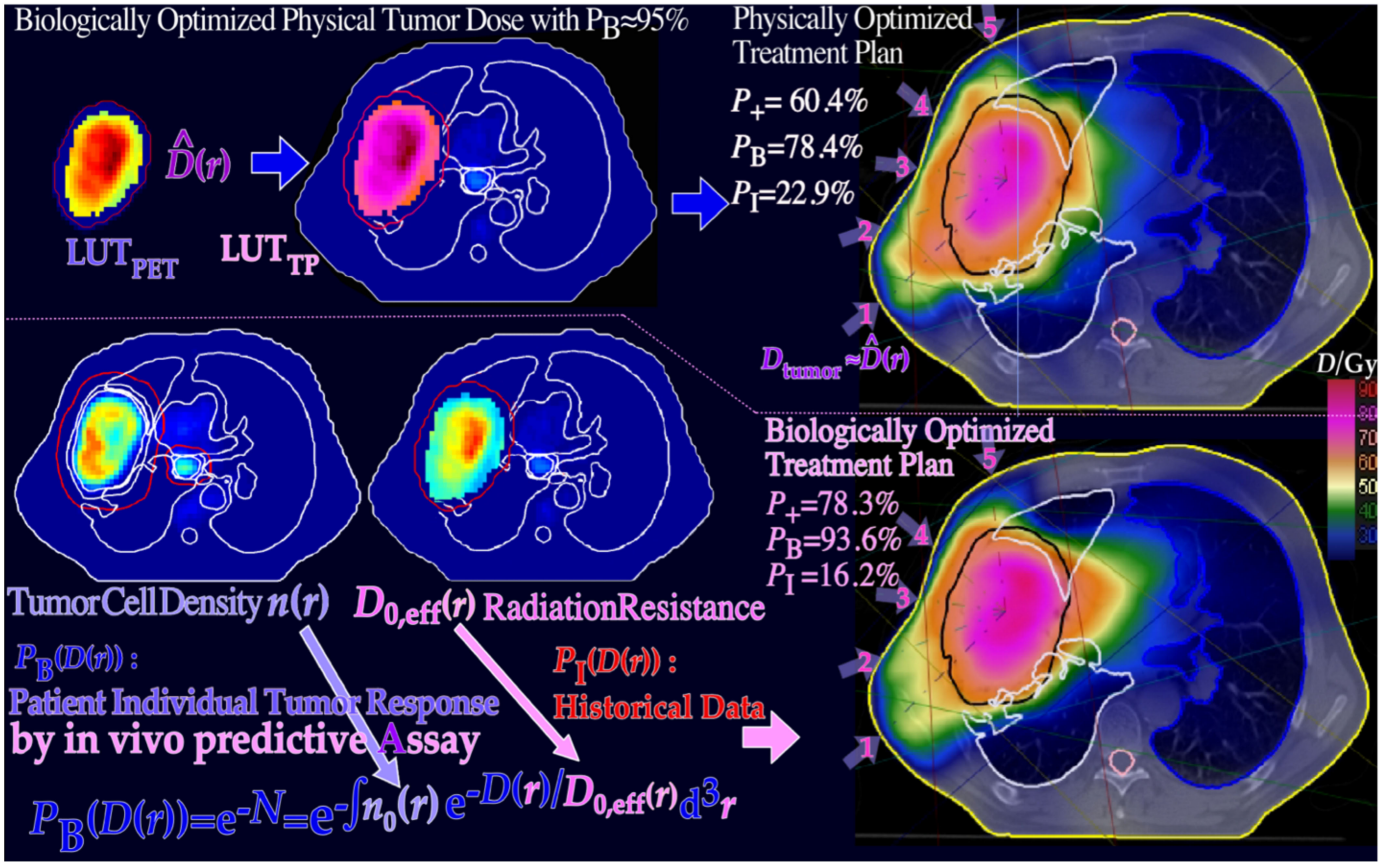
The optimal dose distribution in **Figure 15** can be used as an objective for inverse physical dose therapy optimization (lower left half (9): Figure 127). Even more accurately, the tumor cell density, *n*(*r*), and estimated *D*_0,eff_(*r*) from Figure 127 can be used for biologically effective dose delivery optimization using initial clinically observed tumor responses and historically observed normal tissue dose–response data. By this approach, the complication-free cure increases from 60% to almost 80% as seen in **Figure 16** by introducing the biological optimization method based on the 3D *in vivo* predictive assay and even one step further by optimal time dose fractionation (cf [7].: Figures 47, 82, and 84 and Eq. (40) (1)), and it is most ideal for molecular radiation therapy with a few lithium ion beams (Figure 8, cf [7].: sections 5–8, 10). The figure is modified from (3, 9) with permission [cf. 7: Figure 128].

In the last 30 years, the field of cellular and molecular radiation biology has developed substantially, and we can today describe the response of heterogeneous tumors and organized normal tissues to radiation therapy quite well as seen above. An increased understanding of subcellular and molecular responses leads to a more general systems biological approach to radiation therapy and treatment optimization (4, 11, 27, 69–72, 79). It is interesting that most of the characteristics of the tissue infrastructure, such as the genetic makeup (cf. Figure 8 and (2): Figures 1 and 2) as well as the vascular system and the nutritional and the degree of hypoxia (cf [2].: section 7), need to be considered to obtain an accurate description of tumor and normal tissue responses to ionizing radiation as indicated in **Figure 15**. A brief description of some of the most important concepts and processes is possible, starting from the key functional genomics pathways of the cell that are responsible not only for tumor development but also for the response of the cells to cellular damage such as radiation therapy. Some of the key mechanisms for cellular damage and damage repair are discussed above, and the key question is how these repair processes can be brought to interact and to inactivate the tumor without severely damaging surrounding normal tissues. It is possible by suitable radiation modalities, such as electrons, light ions, and photons, to use biologically optimized radiation intensity- and quality-modulated radiation therapy [IMRT and QMRT (11, 20, 71)] as indicated in **Figure 16**. Interestingly, the clinically observed tumor response after the first week of therapy (cf [2].: Figure 123, lower left panel) and historically observed normal tissue dose–response data for normal tissue side effects are systematically much more similar between patients than the effective tumor response that can vary substantially (cf [2].: Figure 124 [71]).

**Figure 17:**
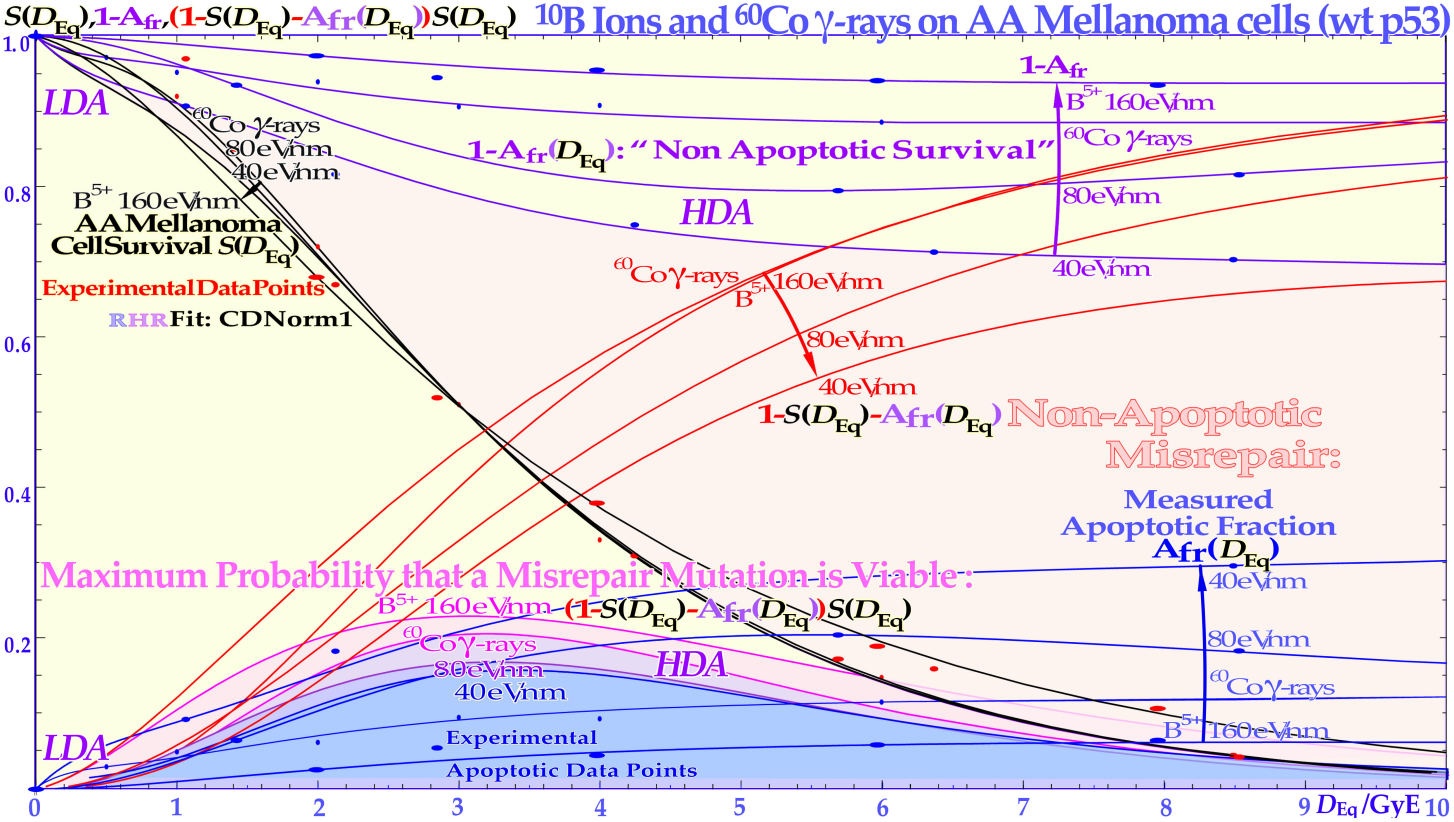
The lower shaded bell-shaped regions show the maximal secondary cancer induction probability as a function of the *LET* and dose equivalent per fraction delivered to tissue. At low doses, the risk of inducing a mutation is small, whereas at high doses, the probability of generating a mutation is higher, but so is the probability of also eliminating it via the treatment. The risk is highest around ≈3 GyE/Fr; thus, this volume in the patients´ normal tissues should truly be minimized. The LDA and LDHS of this TP53 intact tumor cell line are clear from the nonapoptotic and survival curve shapes for the two lowest *LET* beams (^60^Co and 40 eV/nm) as they practically coincide at low dose equivalents (black and violet curves). Interestingly, the risk is the smallest for the lowest-*LET* boron ions due to their high LDA and HDA. The middle shaded area is due to nonapoptotic misrepair for 40 eV/nm ^10^B ions as also shown by the red nonapoptotic misrepair curves. All data points are experimental and the RHR formula is used with the CDN1: one-dimensional closest distance norm, for the fit to data [not least square; for details (7, 9, 10)]. The figure is modified from (7, 9) with permission [cf. 7: Figure 119].

## 8 Low and high *LET* secondary cancer risks

**Figure 18:**
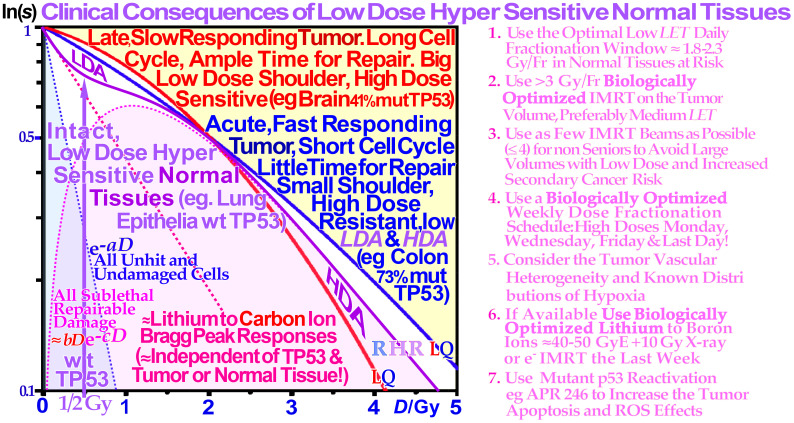
Close-up view of the characteristic cell survival curves of intact LDHS normal tissues (violet), whereas most tumor cell lines often have a mutant TP53 gene, resulting in a low-dose radiation-resistant (LDRR) phenotype due to reduced or lost LDA (cf. Figures 8, 9, and 13). As seen from the low-dose curve shapes, for effective cure, such tumors generally benefit from the lightest ions with the lowest possible *LET* in normal tissues or high IMRT doses with low *LET* especially on slowly responding tumors. On the right side, some of the key clinical conclusions drawn from these curve shapes are summarized (cf [1, 3, 5, 7]. for further details). In radiation therapy, this means that the fully functional DNA repair system should continue to be utilized until more severe HDA sets in beyond 2–2.5 GyE. Thus, there is a low-*LET* optimal radiation therapy fractionation window in normal tissues ≈ 1.8–2.3 Gy/Fr to minimize normal tissue damage, as seen indirectly in **Figures 8** and **13** and here, as further discussed in detail in ((7): Figures 57, 58, 77, 78, 82, 84, and 100 cf. also section 5.5, 5.6 [3, 7, 90]). The structure of high *LET* ion survival is also indicated as the low *LET-*induced LDA and HDA regions of tumors and normal tissues practically overlap (1–1.5 Gy ions ≈ 3–5 GyE) as clearly seen by the detailed apoptosis data in **Figures 10** and **17** (see also [7]: Figures 45, 46, 97, 100–103, and 119). Modified from [7: Figure 140].

The new power of being able to quantify apoptosis from cell survival data (7) makes it possible to better estimate the probability of inducing a secondary cancer especially with experimental cell survival and apoptosis data as shown in **Figure 18** based on the experimental data in **Figure 10** [cf [7].: Figure 6 and (2) Eq. (10)]. It is unlikely that the apoptotic fraction will contribute to secondary cancer induction (except possibly in TP53 mutant cell lines that may integrate DNA fragments from apoptotic bodies into their genomes)!, so it is useful that this fraction can be estimated using the new RHR formula and removed from other forms of misrepair to more accurately describe the cells that are potentially capable of generating a secondary cancer. This cell fraction, as shown in **Figure 17**, has its secondary cancer induction peak in the 3 GyE/Fr region; thus, in radiation therapy optimization, it is truly desirable to minimize this volume in normal tissues as much as possible. **Figure 18** also shows that the maximal risk is the smallest for low-*LET* ions (blue-shaded, 40 eV/nm), largely due to their high apoptotic fraction induction. The real secondary cancer risk may be on the order of 5% of the maximal values in **Figure 17** or less. Obviously, these experimental data are not truly relevant for all surrounding normal tissues that may receive a fair dose and are at risk for a secondary cancer. However, the present tumor cell line is at least wt TP53, so probably not the most extremely mutated one and can, in a first approximation, be assumed to be representative for both normal and tumor tissue at risk. Furthermore, the dose axis is clearly the dose per fraction, so it means that the total dose is increased by the number of fractions used.

Interestingly, the plot is drawn as a function of dose equivalent (Dose × RBE; the 50% survival RBEs used are given in the figure), so all the maximal induction doses align very well, indicating that the ion with the lowest *LET* and highest apoptosis will always minimize the secondary cancer risk for a given delivered dose equivalent. In fact, just as nature arranged it with the LDA to avoid cancer before full repair is induced (cf [7] section 4.7 and presented here in **Figures 8** and **13**), but this time, HDA is also involved (7). Notably, this secondary cancer risk is a contraindication for large, low-dose volumes with many beam portals in intensity-modulated photon therapy using methods such as “rapid arc”, “volumetric arc”, and “tomotherapy” for nonseniors, which may have time to develop secondary cancers 15–20 years after the treatment (72, 79, 80). Interestingly, the new fractionation procedure proposed above (cf [7]: section 5.5) and the new treatment approach suggested in **Figure 12** will allow fewer beam portals and higher tumor doses with fewer adverse reactions in normal tissues using ordinary fixed beam delivery. In fact, if minimal risk for secondary cancer is also a goal of the treatment, multiportal molecular radiation therapy with lithium ions (cf. Figure 8) with half to one GyE plateau dose/portal would be ideal, as seen in **Figure 17**. With the lightest ions, this is generally a smaller problem, as fewer beam portals are needed, and it should thus be the treatment of choice for nonseniors that hopefully will recover from a well-optimized local treatment with minimal risk of late morbidity!

## 9 Low-dose hypersensitivity and apoptosis and light ions in radiation therapy optimization

The new understanding that most normal tissues with an intact TP53 gene are LDHS as discussed above (cf. Figures 7, 8, and 14 [1, 3, 7]) means that the tumor dose per fraction should no longer be 2 Gy, since this is in the region where tumor and normal tissues are almost equally sensitive as seen in **Figure 18**. It is then better to truly use the fractionation window advantage and use the radiation resistance induced by the first 1/2 Gy and continue up to 1.8–2.3 Gy after which HDA sets in. This means that the tumor dose per fraction will be >3 Gy, which is good, especially for the slowly responding tumors, as shown in **Figures 5**, **9**, **12**, and **18**. Assuming that the tumor is mutant on TP53, as most tumors are, we can then use the classical LQ model (otherwise, we need the 3rd RCR or 4th RHR formulation in (9): Figure 49 such as for the melanoma in Figures 45, 101, 103, and 119, cf. also present Figures 10 and 17). However, to avoid normal tissue damage as far as possible, the lightest ions from helium to boron are most effective since their ionization density is mainly elevated in their Bragg peaks to be solely placed in the gross tumor and allowing the effective use of the clinically low *LET* well-established LDHS and LDA fractionation-window at ≈2 Gy/Fr in normal tissues (see (9): section 4.7). **Figure 12** and **18** thus implies that the low *LET* tumor dose should generally be well above the 2-Gy level to ensure an effective cure! In fact, the 2-Gy dose level approximately harms both tumors and normal tissues to the same extent. The differences in the wide spectrum of TP53 mutations and the active gene pool of their host tissues will likely determine and influence the amount of the remaining LDA and HDA of different tumor cell lines largely in agreement with the results of the recent interesting studies (81, 82). In fact, the clinical use of high doses per fraction is now more active than ever (77, 83–95). The recent developments sometimes call this tumor dose escalation “ultra-hypo-fractionated” radiotherapy partly due to the lack of full understanding of the low *LET* fractionation window mechanism (1, 9, 15, 77, 83–95). Interestingly, the optimal dose is determined by the 1.8-2.3 Gy/Fr to organs at risk (cf [7].: Figure 81) and the maximum tolerated tumor dose per fraction as determined by the maximum amount of tumor cell kill per fraction accepted and may partly be determined by the efficiency of the IMRT approach employed! The TP53 gene and its key associated DNA repair pathways, NHEJ and HR, are linked to the LDHS and low-dose apoptosis of most normal tissues, whereas most experimental tumor cell lines are rather radiation-resistant at low doses often due to a mutant TP53 pathway, as shown in **Figure 8** (lower left insert), 9, and 18. This has important consequences for radiation therapy (and radiation protection for that matter) as it causes the well-known clinical fractionation window with minimal damage and apoptosis in normal tissues at ≈2 Gy/Fr of low-ionization-density radiations as shown in (7): Figures 50, 81, and 82. The common tumor radiation resistance (LDRR, see **Figure 9**) at low doses due to TP53 mutations can be treated most effectively by light ions as seen in **Figure 12** but also using p53 reactivation (cf [7].: section 9, Figures 120 and 121). However, to avoid normal tissue damage, the lightest ions from helium to boron are most effective since their ionization density is mainly elevated in their Bragg peaks to be solely placed in the gross tumor and allowing the effective use of the clinically well-established low *LET* fractionation window.

Based on the above-described new treatment optimization principles, we can now state the key goals of advanced radiation therapy more clearly and simplistically be formulated as condensed in the following explanatory clinical conclusions:

1. The peak absorbed dose to critical normal tissues with adverse reactions when quasi uniformly irradiated (≈ organs at risk) should preferably be in the range from 1.8 to 2.3 GyE/Fr and of the lowest possible *LET* and biological effectiveness (cf. (7): Figures 47, 49, 81, and 82). Interestingly, this is the dose and *LET* range that maximizes the LDHS-related normal tissue tolerance with wt TP53 as seen in **Figure 14** (3, 7, 10, 58, 71, 79). A full minimization of the total risk for complications would naturally be preferred and even a full so-called *P*_++_ optimization strategy approach combining conclusion 1 with conclusions 2 and 4 below considering the specific sensitivity of the organs at risk (cf. also Figures 1, 7, and 16 [15, 16]).
2. In order to make the treatment as curative as possible, it is desirable that the mean dose to the tumor is as high as possible without breaking conclusion 1 and not causing massive necrosis in order to ensure true complication-free cure (*P*_+_) and perfect clonogenic tumor cell eradication in the internal target volume (9). Interestingly, this can be achieved quite accurately today by advanced biologically optimized intensity modulated radiation therapy from a few inversely planed beam directions (4, 72, 79) using the appropriate cell survival formulation (cf [7].: Figures 49–53). This will work well even for intact TP53 and ATM pathway tumors (cf. Figures 10 and 17) since a simple LQ-type calculation may be far from optimal for the response of most normal tissues!
3. To further minimize normal tissue damage as far as possible, it is desirable to introduce an optimal daily and weekly dose fractionation schedule where the DNA repair in normal tissues is seriously taken into account to minimize their injury and maximize repair. Up towards 50% higher tumor doses may be delivered on Monday morning, Wednesday midday, Friday evening, and the last evening of treatment, to use the weekend and end of therapy for maximal normal tissue recovery (see **Figure 14**) and preferably still staying below the 2.3 Gy/Fr to organs at risk. This will especially optimize the weekly HR recovery towards ≈ 72+ h (cf. also Figure 14 insert), since NHEJ does it quite well in the 24+ h from day to day. This fractionation advantage works well for low *LET* radiations but also for the lightest ions with mainly a low *LET* in normal tissues!
4. For elderly patients, a larger number of optimized beam portals may be ideal, whereas younger patients may benefit from fewer beams (<5) and a medium *LET* (see section 8) to reduce the risk for secondary cancers in extended low-dose regions after ≈15 years (cf. Figure 17 [4, 73, 78]). These volumes should therefore be reduced as far as possible by sharp penumbras (21) and no arc therapy (80), simultaneously as the complication-free cure (*P*_+_) or preferably the *P*_++_ optimization strategy (*P*_+_ followed by a constrained injury relaxation) are the key objectives of the treatment ((11): Figure 22).
5. To further increase the biologically effective tumor dose delivery, a few light ion beam portals preferably in the range from helium to boron ions only with their Bragg peaks located in the gross tumor volume, to keep a low *LET* (<5 eV/nm) and the dose within 1.8–2.3 Gy/Fr in organs at risk (**Figures 13** and **14** (3, 9)). Organs at risk have to be passed through by beams to reach the target volume, and with the lightest ions (He-B), this can be done by a fairly low *LET* (<5–10 eV/nm). To maximize the complication-free cure with carbon or heavier ions, it is best to switch to electrons or photons in the last 10–15 GyE, and for bulky tumors, preferably use a light ion concomitant gross tumor boost in the last 5 GyE before the final plain 10 GyE low *LET* roundup (Figures 11 and 12, cf [7].: Figure 80 [2, 7];).
6. Consider the tumor vasculature heterogeneity and the distribution of hypoxia. It was carefully calculated for several key tumor types and showed good agreement with clinically measured Eppendorf distributions of hypoxia as seen in (9): Figure 113. This clinically very useful data set for treating common hypoxic tumors by low *LET* later showed that the optimal *LET* for treating them is only as low as 25 eV/nm ((7): Figure 114). This is in good agreement with the optimal *LET* window of 15–55 eV/nm (cf. Figure 17 [7]: Figure 106 [57]); thus, it can also cover other types of tumor heterogeneity and radiation resistance by helium to boron ions.
7. For the multitude of radiation-resistant TP53 mutated tumors that often are a severe clinical problem, the interesting p53 reactivating PRIMA-1 or APR-246 compounds may be useful to increase tumor cell apoptosis and further augment the radiation-induced reactive oxygen species effects in the high tumor dose volumes (7). Interestingly, PRIMA-1 and APR-246 promote the normal function of a missense mutant p53 protein increasing LDA- and HDA-induced apoptosis in the tumor as well as senescence (cf [2].: Figures 120 and 121 [1, 8, 59, 96–102]). Among other effects as seen in (9): Figure 120, it inhibits the enzyme thioredoxin reductase 1 and thioredoxin and decreases cellular glutathione levels, which is especially valuable with low *LET* radiations with higher level of reactive oxygen specis, especially when the lightest ions are not available as seen in (7): Figure 121 (1).

Taking the above approaches into account, the resultant increase in complication-free cure is likely to reach improvements by as much as 30% and more (7, 102). Approximately half of this improvement alone was estimated to come from the improved fractionation schedule discussed in (9): sections 5.5 and 5.6 and Figures 82–84 (8) and equally important is to use a 10-Gy low *LET* roundup in carbon ion therapy to gain steepened dose response, minimize normal tissue damage, and maximize complication-free cure (*P*_+,_*P*_++_).

## 10 Conclusions

Contrary to the common belief that simple DSBs are potentially lethal, we have shown that the plain DSBs are repaired to 99% at 2 Gy, and that the common effectors of curative radiation therapy are the DDSBs at the periphery of nucleosomes that often induce lethal tumor damage beyond approximately 2 Gy low *LET*. The most important clinical consequence is that we need to reconsider the classical 2 Gy/Fr mean tumor dose for low-*LET* radiation based on the molecular processes that make most normal tissues truly LDHS with LDA and HDA due to intact TP53 genes. Thus, full NHEJ and HR repair activity is induced in normal tissues after 0.5 Gy, and therefore at 2 Gy with ≈1.5 Gy being delivered with the clinical advantages of fully efficient NHEJ and HR repair and almost full NHEJ recovery before the next day’s treatment fraction. To minimize damage to normal tissues at risk, a maximum dose of 2.3 Gy/Fr (or a little less) implies optimal tolerance of many normal tissues and allows a significant tumor dose per fraction boost and approximately a 10- to 15-Gy total absorbed dose reduction, cf. Figures 11 and 12 [7]. This also means avoidance of the more severe HDA that sets in after 2–3 GyE. To truly introduce a major paradigm shift in curative radiation therapy thinking, the time dose fractionation should also be optimized for optimal weekend HR recovery (≈50% and complete NHEJ recovery) in normal tissues by higher doses on Friday evening and Monday morning and midday Wednesday. Furthermore, the high microscopic heterogeneity implies that optimal use of carbon ions is with a 10- to 15-Gy low-*LET* treatment roundup to ensure the highest possible dose–response steepness, minimal normal tissue damage, and maximum complication-free tumor cure! To regain the low-*LET* fractionation window at 1.8-2.3 Gy/Fr in normal tissues, we urgently need to open the door for lightest ions ≈ helium–lithium, allowing the more efficient apoptotic–senescent Bragg peak molecular radiation therapy approaches as shown in **Figure 6**, which are maximized in the tumor and minimized in normal tissues. The fractionation window is one of the most important lifelines of curative radiation therapy as proven over more than a century of successful treatments and the more recent molecular and analytical understanding as shown in **Figures 8**–**14** (1, 7–9, 12–16). Taking the above approaches into account, the resultant increase in complication-free cure will likely improve by as much as 30% for many tumor sites. For example using the weekly fractionation scheme and the mutant p53 reactivating compounds [e.g., APR-246 (1, 7, 9, 15, 16, 102)]. Approximately half of this improvement came from the fractionation schedule (**Figures 11**–**14**, **18**) as well as from a better understanding of DDSBs, LDHS, LDA, HDA, and LDRR (**Figures 8**, **9**, **18**) and the optimal use of light ions as shown in **Figures 10**–**14** (7, 9, 10, 15).
